# Spectral characterization of human leg EMG signals from an open access dataset for the development of computational models

**DOI:** 10.1371/journal.pone.0302632

**Published:** 2024-04-29

**Authors:** Roberto Martins de Freitas, André Fabio Kohn

**Affiliations:** 1 Department of Neurological Surgery, University of Pittsburgh, Pittsburgh, Pennsylvania, United States of America; 2 Rehabilitation and Neural Engineering Laboratory, University of Pittsburgh, Pittsburgh, Pennsylvania, United States of America; 3 Biomedical Engineering Laboratory, EPUSP, University of São Paulo, São Paulo, Brazil; Università degli Studi di Milano: Universita degli Studi di Milano, ITALY

## Abstract

Large-scale neuromusculoskeletal models have been used for predicting mechanisms underlying neuromuscular functions in humans. Simulations of such models provide several types of signals of practical interest, such as surface electromyographic signals (EMG), which are compared with experimental data for interpretations of neurophysiological phenomena under study. Specifically, realistic characterization of spectral properties of simulated EMG signals is important for achieving powerful inferences, whereas considerations should be taken for myoelectric signals of different muscles. In this study, we characterized spectral properties of surface interference pattern EMG signals and motor unit action potentials (MUAP) acquired from three plantar flexor muscles: Soleus (SO), Medial Gastrocnemius (MG), and Lateral Gastrocnemius (LG); and one dorsiflexor muscle: Tibialis Anterior (TA). Surface EMG signals were acquired from 20 participants using the same convention for electrode placement. Specifically, interference pattern EMG signals were obtained during isometric constant force contractions at 5%, 10% and 20% of maximum voluntary contraction (MVC), whereas surface MUAPs were decomposed from surface EMG signals obtained at low contraction forces. We compared the spectrum median frequency (MDF) estimated from interference pattern EMG signals across muscles and contraction intensities. Additionally, we compared MDF and durations of MUAPs between muscles. Our results showed that MDF of interference pattern EMG signals acquired from TA were higher compared to SO, MG, and LG for all contraction intensities i.e., 5%, 10%, and 20% MVC. Consistently, MUAPs acquired from TA also had higher MDF values and shorter durations compared to the other leg muscles. We provide herein a dataset with the surface MUAPs waveforms and interference pattern EMG signals obtained for this study, which should be useful for implementing and validating the simulation of myoelectrical signals of leg muscles. Importantly, these results indicate that spectral properties of myoelectrical signals should be considered for improving EMG modeling in large-scale neuromusculoskeletal models.

## 1 Introduction

Surface electromyography (EMG) signals have been widely used for assessing different aspects of neuromuscular function [1–4], as well as for diagnosing neurological disorders [5,6]. Large-scale neuromusculoskeletal computational models also use surface EMG signals for predicting neuromuscular responses, which may not be directly assessed through experiments with human subjects [7–11]. Specifically, surface EMG signals are simulated as an output to computational neural models, which reproduce motor control mechanisms of experimental conditions [8,12–15]. For validating and interpretating neural mechanisms simulated with these large-scale computational models, experimental and simulated surface EMG signals must be quantitatively compared [12–15]. Therefore, realistic simulations of surface EMG signals are required for an adequate interpretation of outcomes obtained from simulations of large scale neuromusculoskeletal models.

Large-scale neuromusculoskeletal computational models simulate surface EMG signals as the sum of motor unit action potential (MUAP) waveforms along time [10,12,15]. A sum of many surface MUAPs from different discharging motor units results in signals with stochastic characteristics, which are referred to as surface EMG interference pattern signals [4]. Morphologies of MUAP waveforms and their patterns of discharge determine stochastic properties of surface EMG interference pattern signals [4,16,17], and thus should be carefully modeled [12,18]. Specifically, De Luca and colleagues have demonstrated that the surface EMG power spectrum at frequencies higher than 20 Hz is mostly determined by the morphologies of MUAPs [19]. Consistently, the spectrum median frequency (MDF) of surface EMG interference pattern signals is decreased as a consequence of longer durations of surface MUAPs during muscle fatigue [20–22]. Overall, morphological characterization of surface MUAP waveforms is required for realistic simulations of surface EMG interference pattern signals in large-scale neuromusculoskeletal models.

Characteristics of myoelectric signals (i.e., surface MUAP and EMG interference pattern) may be critically affected by electrode configurations used during recordings [4,9,16,17,23], as well as electrical properties and anatomy of muscle fibers [24–28]. For instance, Farina has shown that surface MUAP waveforms with longer durations are acquired from muscle fibers with longer lengths [16]. Moreover, it has been previously suggested that the relative position between the recording electrodes and the muscles fibers may also influence the morphology of surface EMG signals [16,23], which is likely to vary across recordings at muscles with different sizes and structure [29]. Taken together, mathematical models of surface EMG signals should be implemented based on characteristics of myoelectric signals acquired from specific muscles using conventional electrode configurations, thus enabling meaningful interpretations from comparisons between simulated and experimental data.

Considering the importance of realistic simulations of myoelectric signals for improving interpretation of large-scale neuromusculoskeletal computational simulations, we characterized the spectral content of surface MUAP waveforms and EMG interference pattern signals acquired from ankle plantar flexor (Soleus, Medial Gastrocnemius, and Lateral Gastrocnemius) and dorsiflexor (Tibialis Anterior) muscles. Specifically, we adopted a convention of electrode configuration for recording all surface EMG signals, such that data from different participants could be pooled together for comparisons. We compared the MDFs and durations of surface MUAP waveforms across the different leg muscles. Moreover, we also compared the MDF estimated from surface EMG interference pattern signals acquired during isometric ankle plantarflexion and dorsiflexion contractions at 5%, 10%, and 20% of maximum voluntary contraction force. We hypothesize that the spectral content of surface interference pattern EMG signals and MUAP waveforms would be different across muscles due to the relative position of the recording electrodes and anatomical properties of their respective muscle fibers, such as fiber length and pennation angle [29,30]. We also expect different MDF values across interference pattern EMG signals acquired at different contraction intensities, considering that changes in fiber pennation angle and length are expected with increasing isometric contraction force [30]. Moreover, considering that the morphologies of surface MUAPs determine spectral characteristics of surface EMG signals [9,16,26], we expect consistency between the MDF and duration values estimated from MUAP waveforms with MDF values estimated from EMG interference pattern signals across different leg muscles. Considering electrophysiological constraints in estimating surface MUAP waveforms at high contraction intensities [3], we have limited our analysis to low contraction forces (i.e., up to 20% of maximum force) for enabling that surface MUAP and EMG interference pattern signals to be jointly used to test this hypothesis.

## 2 Methods

### 2.1 Participants

Twenty able-bodied right-footed individuals, twelve males (age 25.50 ± 2.02, mass 73.33 ± 9.15 kg, and height 1.79 ± 0.07 m; mean ± standard deviation) and eight females (age 23.87 ± 3.13, mass 58.75 ± 8.38 kg, and height 1.66 ± 0.06 m; mean ± standard deviation), participated in this study. Participants were recruited from the 31^st^ of July/2018 until the 11^th^ of September/2018. None of the participants had a history of neuropathy or myopathy. Prior to the experimental session, all participants were informed of the experimental procedures and signed a consent document (written consent). The experiments were conducted in accordance with the Declaration of Helsinki and the experimental protocol was approved by the ethics committee of the School of Physical Education and Sports at the University of São Paulo (CA: 86437518.5.0000.5391).

### 2.2 Data acquisition

**Fig 1:**
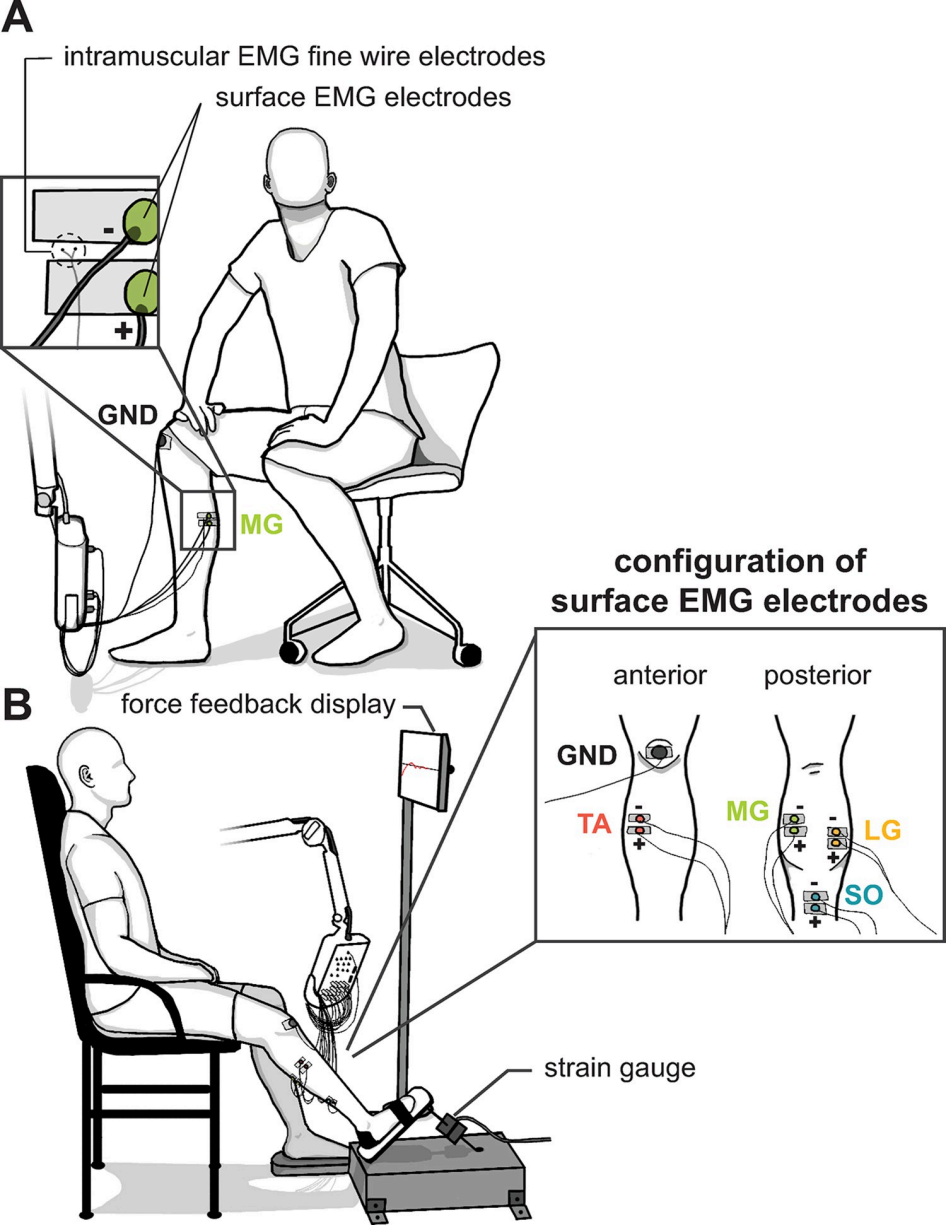
Experimental setup for (A) estimation of surface MUAP waveforms and (B) recording surface EMG interference pattern at different force levels. (A) Experimental setup for estimation of motor unit action potential waveforms from surface electromyography (EMG) signals of four leg muscles: Soleus (SO), Medial Gastrocnemius (MG), Lateral Gastrocnemius (LG), and Tibialis Anterior (TA). Surface EMG signals were recorded, while the participant executed weak contractions. Intramuscular EMG signals were also recorded using two fine wire electrodes inserted in a region between a pair of surface electrodes. (B) Experimental setup for recording surface EMG interference pattern signals. The participant executed isometric plantar-flexion and dorsiflexion contractions at 5%, 10% and 20% of the maximum voluntary contraction force, while the right foot was strapped to a rigid pedal coupled to a strain gauge. Feedback of the force produced on the rigid pedal was displayed on a monitor placed in front of the participant. Surface EMG signals from MG, LG, and TA muscles were acquired using electrodes positioned according to the SENIAM convention, whereas myoelectric signals from SO muscle were acquired using the arrangement adopted in [32]. The most distal electrodes were connected to the “+” terminal of EMG machine, while the most proximal electrodes were connected to the “-” terminal.

**Surface EMG acquisitions.** Surface EMG signals were recorded from Soleus (SO), Medial Gastrocnemius (MG), Lateral Gastrocnemius (LG), and Tibialis Anterior (TA) muscles of the right leg. We used two round Ag/AgCl surface electrodes with 1 cm of diameter, which were distanced by 2 cm along the proximal-distal axis for acquisitions of each muscle. For MG, LG and TA muscles, surface EMG electrodes were positioned according to the *SENIAM* recommendation ([31]; available online in http://seniam.org/sensor_location.htm). The electrodes used for acquisitions of surface EMG signals from SO muscle were conventionally placed 4 cm below the edge of gastrocnemius heads and along the medial line of the leg [32]. A ground electrode with 3 cm diameter was placed over the patella. The electrode configurations for each muscle are illustrated in Fig 1. The skin regions where the surface electrodes were positioned were shaved and abraded with abrasive gel, whereas the electrodes were adhered with conductive gel and fixed with adhesive tape. The surface EMG signals were amplified in differential mode (bipolar acquisition) and band-pass filtered between 10 Hz and 2 kHz using an electromyography system (MEB-2300K, Nihon Kohden, Japan). Moreover, the connection of the electrodes to the electromyography system was standardized: the electrode placed more distally was connected to the “*+*” terminal and the electrode placed more proximally was connected to the “*-*” terminal. The amplified and filtered surface EMG signals were AD-converted at a sampling rate of 20 kHz and a resolution of 16 bits (CED Power 1401, Cambridge Electronic Design Ltd., U.K.).

#### Fine wire EMG acquisitions

Intramuscular EMG signals were recorded from SO, MG, LG, and TA muscles using fine wire electrodes. Specifically, two coated stainless-steel fine wires (A-M Systems, U.S.A.) with 114.3μm of diameter (conductive surface with 50.8μm of diameter) were used. The fine wires were inserted into the muscle region between the pair of surface electrodes using a sterilized hypodermic needle (Fig 1A). The intramuscular EMG signals were amplified in differential mode (bipolar acquisition) and band-pass filtered between 10 Hz and 5 kHz using an electromyography system (MEB-2300K, Nihon Kohden, Japan). The amplified and filtered intramuscular EMG signals were AD-digitalized at a sampling rate of 20 kHz and a resolution of 16 bits (CED Power 1401, Cambridge Electronic Design Ltd., U.K.).

### 2.3 Experimental protocols

EMG signals were recorded from SO, MG, LG and TA muscles in two different experimental protocols for estimation of surface MUAP waveforms: *(protocol 1*) during minimal voluntary contractions, while discriminable MUAP waveforms were acquired from the surface EMG signals; and (*protocol 2*) during weak voluntary contractions, while discriminable MUAP waveforms were acquired from the intramuscular EMG signals. Moreover, we recorded surface EMG interference pattern signals in a third experimental protocol: (*protocol 3*) during isometric plantarflexion and dorsiflexion contractions at 5%, 10%, and 20% of the maximum voluntary contraction force. All the three experimental conditions were conducted in a single experimental session for all participants.

#### 2.3.1 Surface EMG signals recorded with discriminable MUAPs (*protocol 1* and *protocol 2*)

Surface and intramuscular EMG signals were acquired during weak voluntary contractions for enabling estimation of surface MUAP waveforms (*protocol 1* and *protocol 2*). Specifically, participants sat on a chair, as illustrated in Fig 1A, while EMG signals from a single target muscle were recorded during voluntary contractions at minimal (*protocol 1*) or weak (*protocol 2*) intensities. Before the recordings, participants were allowed to freely change the position of the right leg to adjust the contraction intensity of the target muscle. Visual and auditory feedback of the EMG signals were provided through the electromyography system. During the recordings, participants were instructed to sustain a steady muscle contraction, while both legs remained at a fixed position. Surface and intramuscular EMG signals were recorded in three trials with 120 s of duration. Participants were encouraged to change the leg position and contraction strategy between trials, aiming for different MUAPs in the EMG signals [33]. For each participant, EMG signals from only two leg muscles (out of four) were recorded, i.e., surface and intramuscular EMG signals of each muscle (SO, MG, LG, and TA) were recorded from only half of the participants (sample size n = 10).

**Fig 2:**
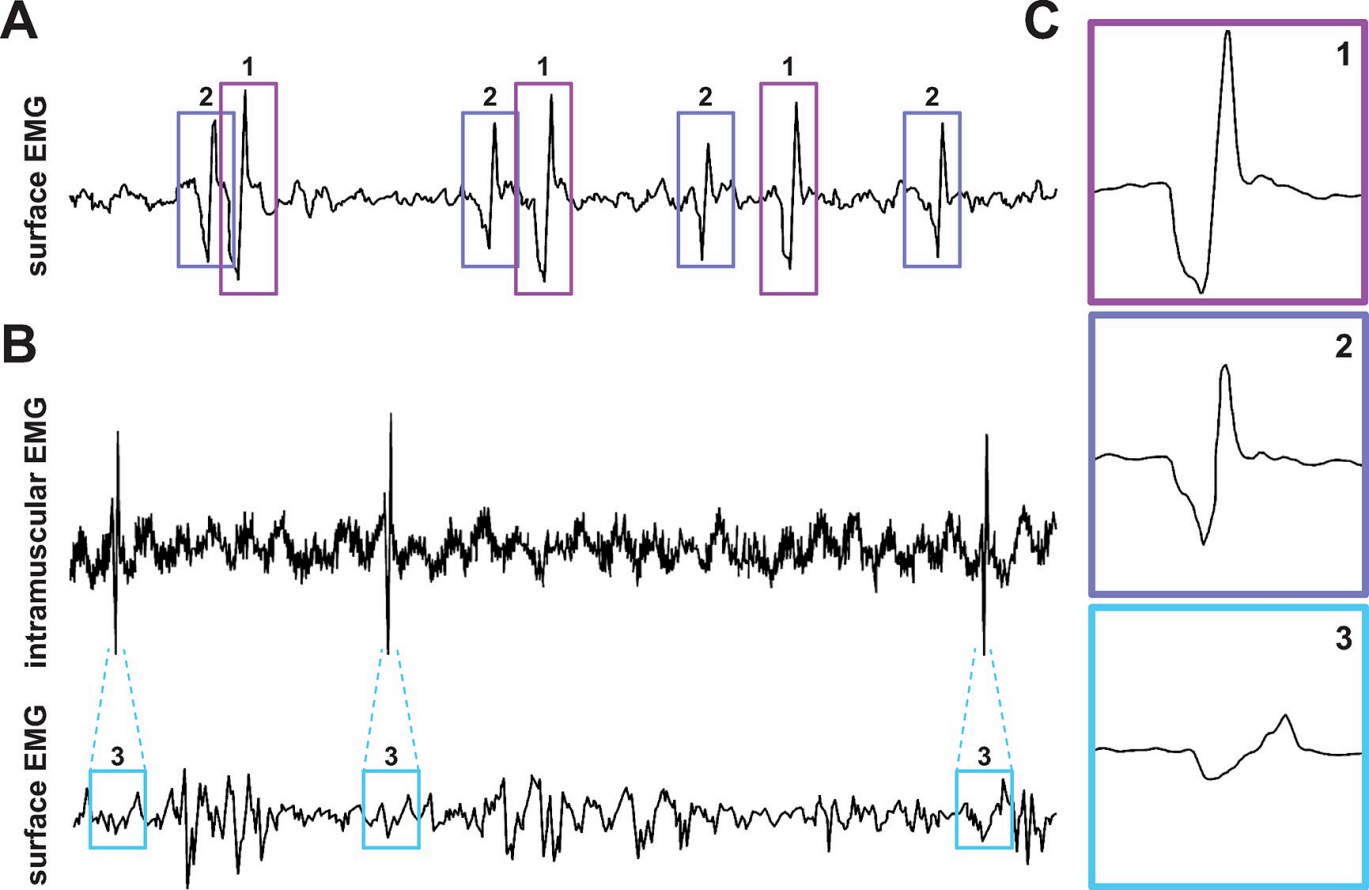
Estimation of surface motor unit action potentials waveforms from the decomposition of (A) surface and (B) intramuscular EMG signals. (A) Surface electromyography (EMG) signal recorded during minimal voluntary contraction. Trains of two surface motor unit action potentials (MUAP) standing out of the signal baseline are identified with rectangles. (B) Surface and intramuscular EMG signals simultaneously recorded during weak contractions. A single motor unit action potential train in the intramuscular EMG signal standing out of the signal baseline is identified, while the surface EMG signal is composed of considerable superpositions of surface MUAPs. (C) MUAP waveforms estimated from the surface EMG signals shown in (A) and (B). The surface MUAP waveforms 1 and 2 were estimated directly from the surface EMG signal using EMGLAB software [34]. The surface MUAP waveform 3 was estimated by averaging the surface EMG signals in time-windows centralized at the peak amplitudes of intramuscular MUAPs.

In *protocol 1*, participants were instructed to sustain a steady voluntary contraction at minimal intensity, while MUAP trains could be discriminated in the surface EMG signals (Fig 2A). Surface EMG signals were recorded when surface MUAPs were clearly standing out of the signal noise baseline. In *protocol 2*, participants were instructed to sustain a steady voluntary contraction at a weak intensity, while surface and intramuscular EMG signals were simultaneously recorded. Fine wire electrodes were inserted not too deep inside the muscle, such that their acquisition volume intersected with that of the surface electrodes, i.e., some MUAPs detected in the intramuscular EMG signals would also be present in the surface EMG signals. In this condition, MUAP trains could be discriminated in the intramuscular EMG signals, while surface EMG signals were composed of sizable superpositions of surface MUAPs (Fig 2B). Surface and intramuscular EMG signals were simultaneously recorded when intramuscular MUAPs were clearly standing out of the signal noise baseline.

#### 2.3.2 Surface EMG interference pattern signals (*protocol 3*)

In *protocol 3*, surface EMG interference pattern signals were recorded during plantarflexion and dorsiflexion isometric contractions at 5%, 10%, and 20% of the maximum contraction force. Specifically, participants sat on an armchair with the right foot strapped on a rigid pedal coupled to a strain gauge (N320, Transtec Ltda, Brazil), as illustrated in Fig 1B. Visual feedback of the force applied to the foot pedal was provided to the participants on a display screen positioned 2 meters away from the armchair. Surface EMG signals from SO, MG and LG muscles were simultaneously recorded during plantarflexion contractions, whereas recordings from TA muscle were performed during foot dorsiflexion. For each subject, the *protocol 3* was executed for plantarflexion and dorsiflexion.

The maximal voluntary contraction (MVC) intensity was measured for determining the target forces intensities (i.e., 5%, 10%, and 20% of the MVC) used during the isometric contraction tasks. The MVC intensity was defined as the maximal contraction force achieved in 3 trials. A resting period of 2 min followed each trial, which had 3 s of duration. Verbal encouragement was given by the experimenter during the MVC task [35]. During the isometric contraction tasks, surface EMG interference pattern signals were recorded from leg muscles while participants applied plantarflexion or dorsiflexion torques at the ankle joint at 3 intensities: 5%MVC, 10%MVC, and 20%MVC. Specifically, participants were instructed to slowly increase the contraction force before reaching the target intensity, and to maintain the contraction force as steady as possible by tracking the visual feedback of the force applied to the foot pedal (Fig 1B). The target intensity was indicated with a red line centered in the middle of the screen and bounded within a ±5%MVC range (e.g. the visual feedback was limited between 5%MVC and 15%MVC in the display screen when the target line was at 10%MVC), such that the feedback visual gain was the same for all participants and contraction intensities [36]. For each contraction intensity, surface EMG interference pattern signals were recorded for 60 s to 150 s.

### 2.4 Signal filtering

All surface EMG signals were band-pass filtered between 20Hz and 1 kHz using a zero-phase *Butterworth* digital filter [19]. In some cases, the EMG signals contained relatively large power line interferences, which corresponded to peaks in the power densities at 60 Hz and its odd harmonics. Therefore, we filtered all surface EMG signals (acquired during all protocols) using zero-phase notch filters at 60 Hz, 180 Hz, 300 Hz, 420 Hz, 540 Hz, 660 Hz, 780 Hz and 900 Hz. The initial and final 5 seconds of each filtered signal were removed due to distortions caused by the zero-phase filtering. Moreover, only the initial 30 s of the surface EMG interference pattern signals were processed and used for subsequent analyses. We did not filter intramuscular EMG signals.

### 2.5 Surface MUAP waveform estimation

Surface MUAP waveforms were detected and estimated from intramuscular and surface EMG signals using EMGLAB software [24,34,37,38]. Specifically, the time instant of peak amplitude of intramuscular and surface MUAPs were automatically identified from EMG signals recorded during *protocol 1* and *protocol 2* (see Section 2.3.1) using EMGLAB [34]. Rigorous manual corrections were performed to group MUAPs with the same amplitude, number of phases and duration. Moreover only MUAPs clearly standing out of the baseline (“noise background”) were considered [24,25]. Surface MUAP waveforms were estimated by averaging time-windows of the surface EMG signals at the time instant of peak amplitude of intramuscular or surface MUAPs, such as illustrated in Fig 2 [24,25,39]. Each MUAP waveform was estimated from at least 100 time-windows, which had a 75 ms duration. It is noteworthy that surface MUAP waveforms averaged with time-synchronized windows from intramuscular MUAPs did not always result in high signal-to-noise waveforms. These low signal-to-noise waveforms were discarded from the analyses. Moreover, when similar surface MUAP waveforms (i.e., similar amplitude, number of phase and duration) were estimated from different recordings (see Section 2.3.1), only the waveform with higher signal-to-noise ratio was considered for analyses. In total, 294 surface MUAP waveforms were estimated (SO: 101, MG: 75, LG: 69, and TA: 49). This dataset of surface MUAP waveforms has been previously used in [24], and it is currently available for use in (https://doi.org/10.17605/OSF.IO/MXTQG).

### 2.6 Data analysis

#### 2.6.1 Duration of surface MUAPs

The durations of the surface MUAP waveforms were obtained from manual measurements done by three researchers of the Biomedical Engineering Laboratory (LEB)–University of São Paulo, who are familiar with surface MUAPs and EMG signals [24,26]. The duration of each MUAP waveform was defined as the average of these three measurements. These MUAP durations have been previously reported in [24], although they were not subjected to statistical analyses.

#### 2.6.2 Spectrum median frequency of surface MUAPs and surface EMG interference pattern signals

The spectrum median frequency (MDF) of surface MUAP waveforms and EMG interference pattern signals were estimated, consistent with [8]. Specifically, the MDF is defined as the value that leads to the identity

$$\sum_{i=1}^{MDF}P_{i}=\sum_{i=MDF}^{M}P_{i}=\frac{1}{2}\sum_{i=1}^{M}P_{i}$$ (1)

where *P*_*i*_ is the value of the EMG power spectrum at the *i*^*th*^ frequency bin, and *M* is the total number of frequency bins [40]. The power spectral densities of both MUAPs and EMG interference signals were estimated with periodograms with 13.333Hz frequency resolution for MUAP waveforms and 0.033Hz frequency resolution for EMG interference pattern signals. The frequency resolutions respectively correspond to 75 ms time-window length used to estimate the MUAP waveforms, and to 30 s length of EMG interference pattern signals for a 20 kHz sampling frequency.

#### 2.6.3 Fatigue effects in surface EMG interference pattern signals

To analyze the effect of muscle fatigue in the surface EMG interference pattern signals, the MDF value along the signal duration was analyzed [17,20,40,41]. It is worth noting that previous studies have shown that a significant decrease in the MDF value of surface EMG signals along time is associated with muscle fatigue [17,20,41]. The power spectral densities were estimated with periodograms of surface EMG signals recorded during *protocol 3* (see Section 2.3.2), consistent with previous studies [17,20,40,41]. For each recording, the MDF values from the first (15 s) and second (15 s) halves were estimated and compared [17,20,40,41]. Data from each muscle (SO, MG, LG and TA) and contraction intensity (5%, 10% and 20% MVC) were pooled together for statistical analyses.

### 2.7 Statistics

To compare the spectral content of surface MUAP waveforms, the duration and spectrum median frequency were compared across SO, MG, LG, and TA muscles. A between-subject design one-way ANOVA was used to compare the duration of surface MUAP waveforms across the different leg muscles (*muscle*: *SO*, *MG*, *LG*, and *TA*). Moreover, a between-subject design one-way ANOVA was used to compare the MDF of surface MUAP waveforms across the different leg muscles (*muscle*: *SO*, *MG*, *LG*, and *TA*).

To compare the spectral content of surface EMG interference pattern signals acquired during isometric contractions at 5%, 10%, and 20% of MVC, the spectrum median frequency was compared across SO, MG, LG, and TA muscles. A between-subject design two-way ANOVA was used to compare the MDF of interference surface EMG signals (*EMG interference pattern*: *EMG*_5%_, *EMG*_10%_ and *EMG*_20%_) across the different leg muscles (*muscle*: *SO*, *MG*, *LG*, and *TA*). Furthermore, effects of muscle fatigue in the surface EMG interference pattern signals were analyzed by comparing the MDF value in the first and second halves of the signals (*half*: *first* and *second*) using paired sample t-test for each muscle (SO, MG, LG and TA) and contraction intensity (5%, 10% and 20% of MVC).

When significant interactions were found on the two-way ANOVA, between-subject design one-way ANOVA tests were conducted for each factor (*signal* and *muscle*). Post hoc multiple comparisons were performed using Bonferroni corrections. The Shapiro-Wilk test was used to test the normality of the data. Although most of the data were normally distributed, non-parametric Kruskal-Wallis test was used to confirm the results obtained with between-subject design one-way ANOVA. All statistical analyses were performed using SPSS (IBM, Armonk, NY) and a significance level *α* = .050 was adopted.

## 3 Results

**Fig 3:**
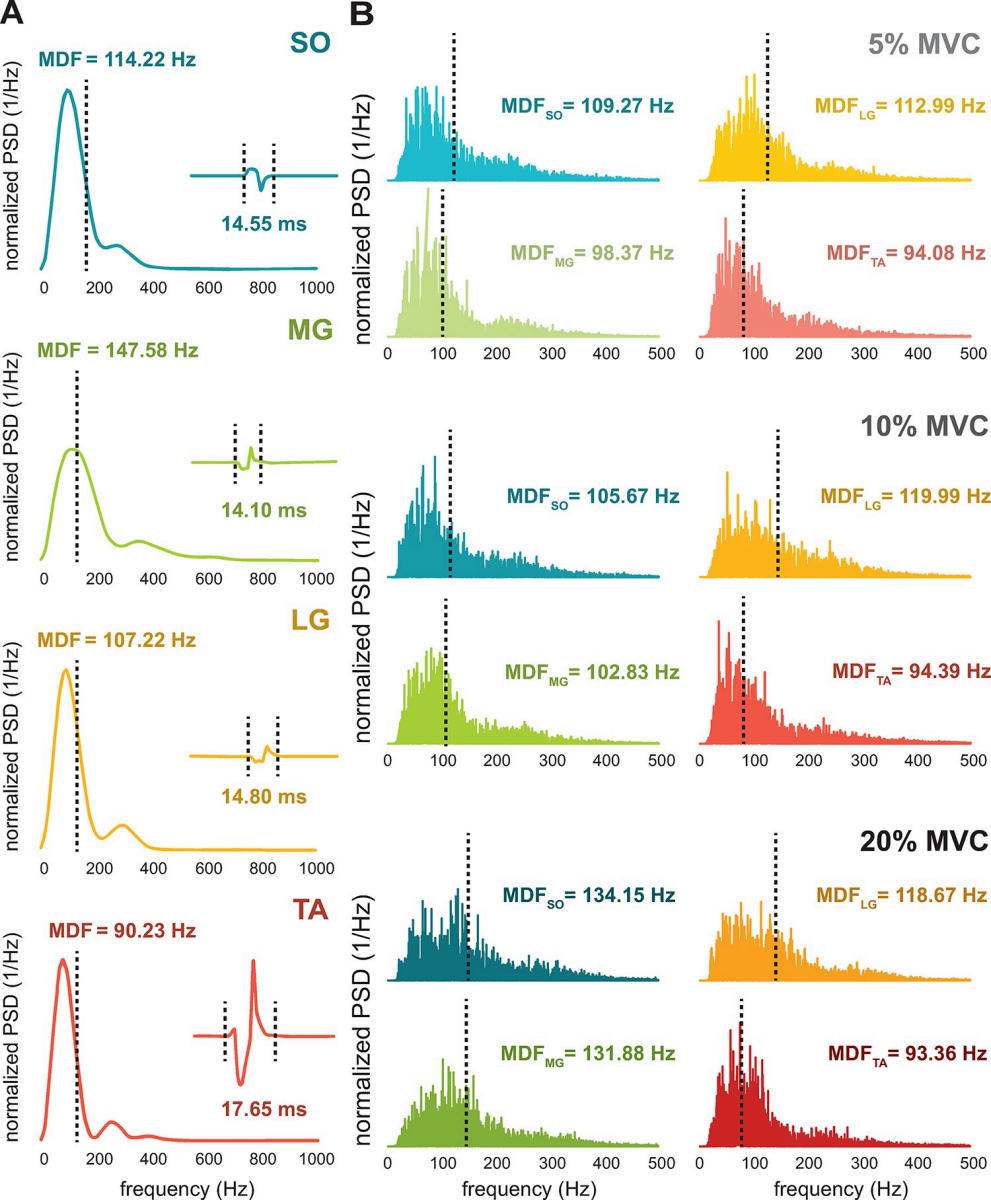
Representative (A) surface MUAP waveforms and (B) EMG interference pattern signals of different leg muscles. (A) Spectrum median frequency (MDF), in Hz, and duration, in ms, of representative motor unit action potential (MUAP) waveforms estimated from surface electromyography (EMG) signals recorded from Soleus (SO), Medial Gastrocnemius (MG), Lateral Gastrocnemius (LG), and Tibialis Anterior (TA) muscles during weak voluntary contractions. The power spectral densities were normalized by the mean square value of the surface MUAP waveform amplitudes. (B) MDF, in Hz, estimated from surface EMG interference pattern signals obtained from SO, MG, LG, and TA muscles of a representative participant during isometric constant force plantarflexion and dorsiflexion contractions at 5%, 10%, and 20% of the maximum voluntary contraction (MVC). The power spectral densities were normalized by the mean square value of the surface EMG signal amplitudes.

We estimated the MDF and durations of MUAP waveforms obtained from surface and intramuscular EMG signals of SO, MG, LG, and TA muscles during weak contractions, as exemplified in Fig 3A. Moreover, we estimated the MDF of surface EMG interference pattern signals acquired muscles during isometric constant-force dorsiflexion and plantarflexion contractions at 5%, 10%, and 20% of MVC, as exemplified in Fig 3B. Taken together, we found that spectral myoelectric signals acquired from TA muscle had more spectral components at lower frequencies compared with the other leg muscles. Consistently, MUAPs obtained from TA had longer durations.

### 3.1 Spectral content of surface MUAP waveforms

In total, we decomposed 294 MUAP waveforms (SO: 101; MG: 75; LG: 69, and TA: 49) from surface EMG signals acquired during low intensity voluntary contractions. We compared the MDFs and durations of the MUAP waveforms across the four leg muscles (*muscle*: *SO*, *MG*, *LG*, and *TA*) using one-way ANOVA. Representative power spectral densities estimated from surface MUAP waveforms obtained from SO, MG, LG, and TA muscles are shown in Fig 3A. In this representative example, the MUAP waveform obtained from TA muscle had the longest duration (17.65 ms) compared with the other plantar flexor muscles (i.e., SO, MG, and LG). Moreover, the MDF was lower for TA (90.23 Hz) compared with those other muscles.

**Fig 4:**
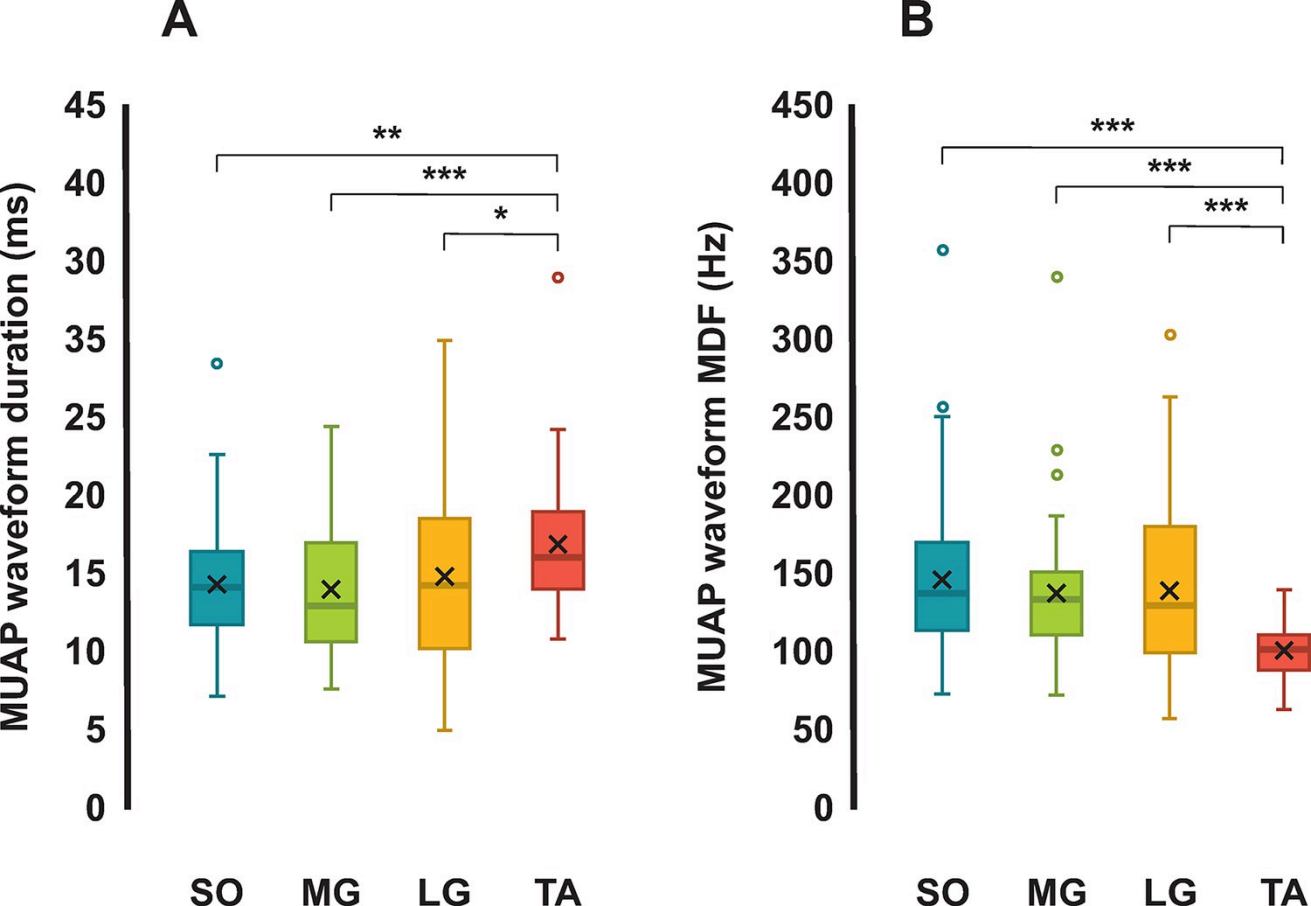
(A) duration and (B) spectrum median frequency of surface MUAP waveforms compared across leg muscles. (A) Box plots of durations, in ms, of the decomposed motor unit action potential (MUAP) waveforms obtained from Soleus (SO), Medial Gastrocnemius (MG), Lateral Gastrocnemius (LG), and Tibialis Anterior (TA) muscles. (B) Box plots of spectrum median frequencies (MDF), in Hz, of the decomposed surface MUAP waveforms obtained from SO, MG, LG, and TA muscles. The Kruskal-Wallis test was used to compared the MDF and durations across the four leg muscles (SO, MG, LG, and TA). The sample size for SO is n_SO_ = 101, for MG is n_MG_ = 75, for LG is n_LG_ = 69, and for TA is n_*TA*_ = 49. The horizontal bars indicate statistical differences across muscles. Legend: * p < .05, ** p < .01, and *** p < .001.

**Durations of surface MUAP waveforms**Results obtained from the comparisons of the duration estimated of surface MUAP waveforms from all subjects, obtained from different muscles (*muscle*: *SO*, *MG*, *LG*, and *TA*), are summarized in Fig 4A. The one-way ANOVA showed significant main effects across different muscles (*F*(3, 290) = 12.731, *p* < .001; SO– 14.393 ± 3.852 ms; MG– 14.022 ± 4.203 ms; LG– 14.811 ± 5.422 ms; TA– 16.960 ± 4.241 ms; mean ± standard deviation). Multiple-pairwise comparisons between *muscle* are shown in Fig 4A. Importantly, the durations of surface MUAP waveforms obtained from TA muscle were significantly longer compared with those from the other muscles.

#### Spectrum median frequency of MUAP waveforms

Results obtained from the comparisons of the MDF estimated of surface MUAP waveforms from all subjects, obtained from different muscles (*muscle*: *SO*, *MG*, *LG*, and *TA*), are summarized in Fig 4B. The one-way ANOVA showed significant main effects across different muscles (*F*(3, 290) = 12.731, *p* < .001; SO– 147.562 ± 4.534 Hz; MG– 139.504 ± 17.592 Hz; LG– 140.924 ± 6.121 Hz; TA– 102.669 ± 17.592 Hz; mean ± standard deviation). Multiple-pairwise comparisons between *muscle*s are shown in Fig 4B. Notably, the MDFs of surface MUAP waveforms obtained from TA muscle were significantly lower compared with the other plantar flexor muscles.

### 3.2 Spectral content of surface EMG interference pattern signals

We compared MDF across different contraction intensities (*EMG*: *EMG*_5%_, *EMG*_10%_ and *EMG*_20%_) and the four leg muscles (*muscle*: *SO*, *MG*, *LG*, and *TA*) using a between-subject design two-way ANOVA. To analyze effects of muscle fatigue along time, we also compared the MDF from the first and second halves (*half*: *first* and *second*) of the EMG interference pattern signals using paired sample t-tests for each muscle (SO, MG, LG and TA) and contraction intensity (5%, 10% and 20% MVC) separately [17,20,41]. Power spectral densities estimated from surface EMG interference pattern signals obtained from SO, MG, LG, and TA muscles of a representative participant are shown in Fig 3B. For all contraction intensities (5%, 10%, and 20% of MVC), the MDF was lower for TA muscle compared with the other plantar flexor muscles (SO, MG, and LG). It is noteworthy that the MDF did not largely change across contraction intensities for all muscles.

#### Spectrum median frequency of surface EMG interference pattern signals

**Fig 5:**
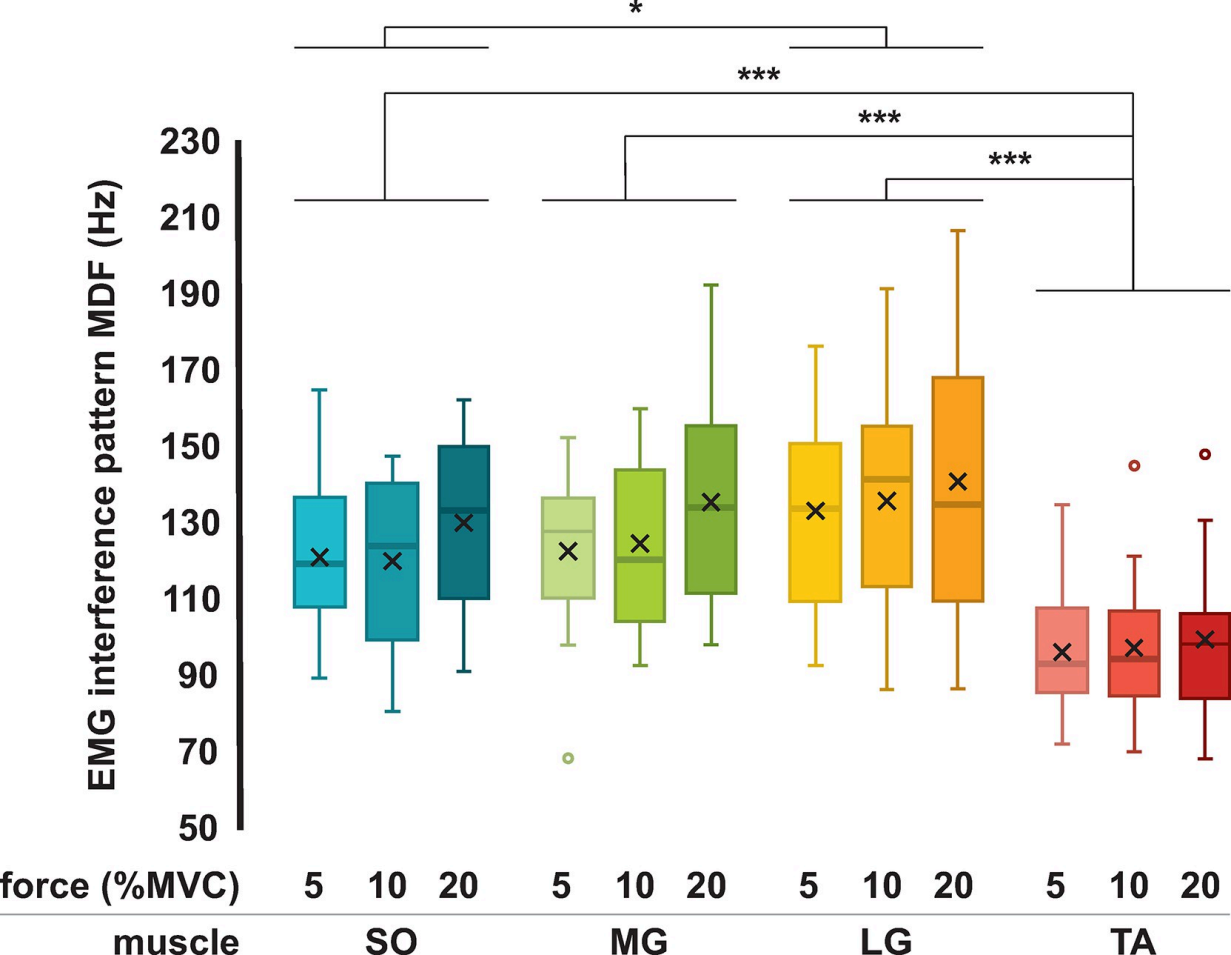
Spectrum median frequency of surface EMG interference pattern signals compared across leg muscles. Box plots of spectrum median frequencies (MDF), in Hz, estimated from the surface electromyography (EMG) interference pattern signals acquired from Soleus (SO), Medial Gastrocnemius (MG), Lateral Gastrocnemius (LG) and Tibialis Anterior (TA) muscles during isometric constant-force plantarflexion and dorsiflexion contractions at 5%, 10%, and 20% of maximum voluntary contraction force (MVC). The standard errors are indicated with vertical bars. The two-way ANOVA was used to compare the MDF across muscles (SO, MG, LG, and TA) and contraction intensities (5%, 10%, and 20% of MVC). The horizontal bars indicate statistical differences across muscles. The sample size for each muscle and contraction intensity is n = 20. Legend: * p < .05, ** p < .01, and *** p < .001.

Results comparing the MDF estimated from surface EMG interference pattern signals recorded from different muscles (*muscle*: *SO*, *MG*, *LG*, and *TA*) at different contraction intensities (*contraction intensity*: *EMG*_5%_, *EMG*_10%_ and *EMG*_20%_) are summarized in Fig 5. The two-way ANOVA showed no interactions between two factors (*F*(6, 228) = .219, *p* = .970), whereas significant main effects were found for *muscle* (*F*(3, 228) = 31.546, *p* < .001; SO– 5%: 121.360 ± 18.575 Hz, 10%: 120.326 ± 21.166 Hz, 20%: 130.306 ± 23.032 Hz; MG– 5%: 122.909 ± 20.934 Hz, 10%: 124.939 ± 20.248 Hz, 20%: 135.722 ± 27.958 Hz; LG– 5%: 133.467 ± 23.030 Hz, 10%: 136.052 ± 25.860 Hz, 20%: 141.139 ± 35.511 Hz, TA– 5%: 96.545 ± 15.184 Hz, 10%: 97.642 ± 17.980 Hz, 20%: 99.797 ± 19.589 Hz; mean ± standard deviation). No significant main effects were found for *contraction intensity* (*F*(2, 228) = 2.948; *p* = .054). Multiple-pairwise comparisons between *muscle* are shown in Fig 5. Importantly, the MDF of EMG signals obtained from TA muscle were significantly lower compared with the other plantarflexion muscles. Moreover, the MDF values of EMG signals obtained from SO muscle were significantly lower compared with LG.

#### Fatigue effects in surface EMG interference pattern signals

The means and standard deviations of the MDF values calculated for the first and second halves for each muscle (SO, MG, LG, and TA) and contraction intensities (5%, 10%, and 20% of MVC) are shown in Table 1. The t-test showed no difference between the MDF in the *first* and *second* halves of the EMG signals acquired from SO (5%: *t*(19) = 1.517, *p* = .146; 10%: *t*(19) = —.746, *p* = .465; 20%: *t*(19) = .145, *p* = .886), MG (5%: *t*(19) = 1.408, *p* = .175; 10%: *t*(19) = -.785, *p* = .442; 20%: *t*(19) = 1.775, *p* = .092), LG (5%: *t*(19) = -.164, *p* = .872; 10%: *t*(19) = -.636, *p* = .532; 20%: *t*(19) = 1.128, *p* = .274) and TA (5%: *t*(19) = .881, *p* = .389; 10%: *t*(19) = 1.700, *p* = .105; 20%: *t*(19) = 1.662, *p* = .113).

**Table 1:** Mean and standard deviation of the power spectrum median frequencies calculated from the first and second halves of surface EMG interference pattern signals.

Muscle	EMG signal period	5% MVC	10% MVC	20% MVC
**SO**	1^st^ half	121.740 ± 18.819 Hz	119.812 ± 20.013 Hz	130.097 ± 24.364 Hz
	2^nd^ half	120.727 ± 18.230 Hz	120.229 ± 20.138 Hz	130.000 ± 23.497 Hz
**MG**	1^st^ half	123.647 ± 20.375 Hz	125.029 ± 21.121 Hz	136.658 ± 28.962 Hz
	2^nd^ half	122.747 ± 20.702 Hz	126.060 ± 19.210 Hz	134.736 ± 27.219 Hz
**LG**	1^st^ half	133.351 ± 22.402 Hz	135.800 ± 26.121 Hz	143.883 ± 35.856 Hz
	2^nd^ half	133.463 ± 22.851 Hz	136.153 ± 26.373 Hz	141.564 ± 32.224 Hz
**TA**	1^st^ half	96.293 ± 14.424 Hz	99.458 ± 18.221 Hz	100.275 ± 18.896 Hz
	2^nd^ half	95.656 ± 15.349 Hz	98.312 ± 17.695 Hz	99.304 ± 19.383 Hz

Mean and standard deviation (mean ± standard deviation) of the power spectrum median frequencies calculated from power spectra estimated from the first and second halves of surface EMG interference pattern signals acquired from Soleus (SO), Medial Gastrocnemius (MG), Lateral Gastrocnemius (LG), and Tibialis Anterior (TA) muscles during isometric constant-force plantarflexion and dorsiflexion contractions at 5%, 10% and 20% of the maximum voluntary contractions (MVC). The sample size for each muscle and contraction intensity is n = 20.

## 4 Discussion

We quantified the spectral content of surface MUAP waveforms and EMG interference pattern signals acquired using a conventional configuration of electrodes from the four major leg muscles actuating at ankle plantarflexion (SO, MG, and LG) and dorsiflexion (TA). The data were obtained from young healthy subjects exerting low levels of constant isometric force. We showed that surface MUAP waveforms obtained from TA muscle had longer durations, as well as more spectral components at lower frequencies compared with plantar flexor SO, MG, and LG muscles (Fig 4). Furthermore, surface EMG interference pattern signals recorded from the TA muscle during isometric dorsiflexion contractions at 5%, 10%, and 20% of MVC had spectral components concentrated at lower frequencies compared with the other muscles (Fig 5). For all muscles, no significant differences were found between the MDF estimated from surface EMG interference pattern signals recorded during different force levels (5–20%MVC) (Fig 5). Additionally, the MDF values estimated from the first and second halves of surface EMG interference pattern signals were not statistically different for all muscles and contraction intensities, suggesting that our results were not significantly affected by muscle fatigue (Table 1). These results inform important morphological characteristics of myoelectric signals from leg muscles for simulation and validation of surface EMG signals in large-scale neuromusculoskeletal computational models.

### 4.1 Spectral content of surface MUAP waveforms

Our results showed that surface MUAP waveforms acquired from the dorsiflexor TA muscle had longer durations and spectral components at lower frequencies compared with the SO, MG, and LG muscles (Fig 4B). These results are consistent with our hypothesis that spectral content of surface MUAP waveforms would be different across muscles. Furthermore, an inverse relationship between the durations and MDFs of MUAP waveforms was found (Fig 4A), consistent with previous experimental and computational studies [21,22]. It is noteworthy that the duration of surface MUAP waveforms may be affected by electrical properties of muscle fibers, such as the conduction velocity of action potentials along muscle fibers [26]. These results indicate that muscle fibers of the TA muscle might have lower conduction velocities compared with the other plantar flexor muscles (SO, MG, and LG), as suggested by [42]. Nevertheless, there could exist multiple confounding factors, such as the relative position of muscle fibers and the recording electrodes, that could explain the morphological characteristics of surface MUAP waveforms [16].

### 4.2 Spectral content of surface EMG interference pattern signals

On the one hand, the MDF values estimated from surface EMG interference pattern signals acquired from the TA muscle were smaller compared with the other plantar flexor muscles (Fig 5), consistent with the differences found for MDF and durations of MUAP waveforms in Fig 4. On the other hand, MDF values estimated from EMG interference pattern signals of the LG muscle were significantly higher compared with those from the SO muscle (Fig 5), which is not consistent with the results obtained from surface MUAP waveforms (Fig 4). Surface MUAP waveforms were estimated from EMG signals acquired at very weak contraction intensities, whereas EMG interference pattern signals were acquired during isometric contractions at up to 20% of MVC. It is thus possible that a change in fiber pennation angle and length with increasing isometric contraction force has resulted in the spectral content of myoelectric signals obtained from LG shifting towards higher frequencies [30]. In fact, MDF values seem to increase with increasing contraction intensities for all muscles in Fig 5, despite not being a statistically significant difference. It is also noteworthy that the Soleus muscle, located beneath the Gastrocnemii and attached to the Achilles tendon, is at a further distance from the recording electrodes compared to the lateral head of the Gastrocnemius muscle [32]. The relative distance between the surface EMG electrodes and the anatomical position of the muscle fibers could also be a confounding factor in the results shown in Fig 5. Nevertheless, our results demonstrate consistency between the spectral and morphological characteristics of surface MUAP waveforms and interference pattern EMG signals acquired from TA and the other plantar flexor muscles (SO, MG, and LG), in agreement with our hypothesis that morphologies of surface MUAPs would determine spectral characteristics of surface EMG signals.

Muscle fatigue is indicated by a compression of surface EMG signal spectra towards lower frequencies and an increase in surface MUAP durations over time [20–22]. Our results showed that the MDF estimated from the first and second halves of the surface EMG signals were not significantly different, suggesting that the spectrum was not compressed along time as an effect of muscle fatigue [17,20,41]. Moreover, surface MUAP waveforms were obtained from EMG signals recorded during relatively short periods (120 s) of very weak contractions, which were unlikely affected by muscle fatigue effects. Therefore, there is evidence that muscle fatigue effects had no significant influence on our results.

### 4.3 Simulation and validation of surface EMG interference pattern signals

We showed that surface EMG signals acquired from TA muscle have more spectral components in lower frequencies compared with the plantar flexor muscles (SO, MG, LG, and TA), whereas their surface MUAP waveforms had longer durations. These results indicate that characteristics of myoelectric signals acquired from specific muscles should be taken into account for realistic simulations of surface EMGs, which may provide adequate parallels between computational simulation results and experimental data [12–15]. The experimental values estimated in this study (Figs 4 and 5) could help the development and validation of computational models of surface EMG of ankle plantar flexor and dorsiflexor muscles. For the validation of computational models, the MDF values shown in Fig 5 could be used as a reference for confirming the spectral components of surface EMG signals simulated, for example, with volume conduction models [27]. Alternatively, the dataset of surface EMG interference pattern signals obtained in this study (available for download in https://doi.org/10.17605/OSF.IO/MXTQG) could be used to statistically validate the spectra estimated from EMG signals simulated with a model under development by proper time series analyses methods [43]. Importantly, the same configuration of electrodes used herein for recording surface EMG signals (see Section 2.2) should be employed in future experimental studies for enabling validations. For the development of phenomenological computational models of surface EMG signals, the dataset of surface MUAP waveforms obtained in this study (available for download in https://doi.org/10.17605/OSF.IO/MXTQG) could be directly summed at time instants corresponding to motor unit discharges. Alternatively, the time-series data of surface MUAP waveforms can be expanded in Hermite-Rodriguez functions, consistent with the approach used in [44] for intramuscular EMG signals. Finite support wavelets can also be simulated as MUAP waveforms by adjusting their morphologic properties, such as duration, number of peaks, and amplitudes, estimated from the MUAP waveforms of our dataset [24]. Moreover, surface EMG signals can also be simulated as the temporal sum of MUAP waveforms available herein for each muscle, instead of mathematically modeled waveforms, thus avoiding approximations stemming from the mathematical modeling [10,15].

### 4.4 Limitations

A limitation of this study is that we did not analyze surface MUAP waveforms and EMG interference pattern signals for contraction intensities above 20% of MVC, which may limit the interpretation of our findings for signals acquired at stronger force levels. It is noteworthy that the estimation of surface MUAP waveforms from EMG signals acquired using bipolar electrode configuration is unfeasible at high contraction forces due to an excessive superimposition of MUAPs [3]. This electrophysiological limitation restricts our ability to use surface MUAP waveforms jointly with EMG interference pattern signals in our analysis. Nevertheless, Farina and colleagues [39] have previously shown that the volume conductor (e.g., layers skin and fat layers) intrinsic to surface EMG recordings restricts the morphological diversity of surface MUAP waveforms even for late recruited motor units, i.e., when the contraction intensity is increased. Therefore, our findings may extrapolate, to a certain degree, spectral characteristics of surface EMG signals acquired during isometric constant-force contractions at intensities somewhat above 20% of MVC.

## 5 Conclusions

We showed that surface MUAP waveforms estimated from EMG signals acquired using a conventional electrode configuration from dorsiflexor TA muscle had longer durations and more spectral components at lower frequencies compared with plantar flexor muscles (i.e., SO, MG, and LG). Moreover, we also found that surface EMG interference pattern signals acquired during isometric contractions at 5%, 10%, and 20% of MVC from TA had more spectral components at lower frequencies compared with plantar flexor muscles. Taken together, the results from this work (the database and the spectral results) should be helpful for those modeling MUAPs and EMGs for computational simulations (e.g., for large-scale neuromuscular simulators) as they serve as quantifiers for validating the chosen mathematical models.
