# Supplementary material for: Spectral characterization of human leg EMG signals from an open access dataset for the development of computational models
Source: PLoS One. 2024 Apr 29;19(4):e0302632. doi: 10.1371/journal.pone.0302632 (PMC11057972; doi:10.1371/journal.pone.0302632)
Supplement: Attachment — Submitted filename: Response to Reviewers.docx [file pone.0302632.s001.docx]

**Responses to Reviewers: Revision 1 (R1)** **for ‘PONE-D-23-42461’ to the** ***PLOS ONE* – February 2024**

The authors would like to thank the Editor and the Reviewer 1 for their constructive comments and suggestions. They helped us improve our manuscript considerably and meet the journal requirements for submission. Responses addressing specific comments from the Editor and Reviewer 1 are outlined below:

---------------------------------------------

**Responses to Reviewer #1:**

The authors would like to thank the Reviewer 1 for the constructive comments and suggestions.

Comment 1:	*The authors acknowledge the limitation in not analyzing surface MUAP waveforms and EMG interference pattern signals for contraction intensities above 20% of maximum voluntary contraction (MVC). I recommend the authors to better explain the reasons behind this choice.*

Thank you for your comment. Our main objective in this study was to compare the spectra of surface myoelectric signals acquired from different leg muscles using both motor unit action potential (MUAP) waveforms and EMG interference pattern signals. For enabling that MUAP waveforms and EMG interference pattern signals could be jointly used in our analysis to support our findings, we have limited our analysis to myoelectric signals acquired at low contraction intensities, i.e., 20% of the maximum voluntary contraction (MVC) force. Specifically, it is noteworthy that the estimation of surface MUAP waveforms from EMG signals acquired using bipolar electrode configuration is unfeasible at high contraction forces (Merletti & Farina, 2016). As the contraction force is increased, the number of recruited motor units and their discharge frequencies (i.e., rate coding) are increased (Enoka & Duchateau, 2017). At these high contraction forces, surface EMG signals are composed of an excessive sum of MUAPs which are not able to be discriminated. Therefore, considering these limiting constraints for estimating surface MUAP waveforms at high contraction forces and our objectives in this study, we have limited our analysis to surface myoelectric signals acquired at contraction forces below 20% MVC. Nevertheless, as we discuss in the Discussion section of our manuscript (4.4 Limitations), the volume conductor intrinsic to surface EMG recordings restricts the morphological diversity of surface MUAP waveforms even for late recruited motor units (D. Farina, Negro, Gazzoni, & Enoka, 2008). Thus, our findings may extrapolate to spectral characteristics of myoelectric EMG signals acquired at contraction forces somewhat above 20% MVC.

The authors acknowledge that these technical constraints must be added to the manuscript for better understanding of the limitations of the study. We have, therefore, added the following considerations to the Discussion section (4.4 Limitations):

" A limitation of this study is that we did not analyze surface MUAP waveforms and EMG interference pattern signals for contraction intensities above 20% of MVC, which may limit the interpretation of our findings for signals acquired at stronger force levels. It is noteworthy that the estimation of surface MUAP waveforms from EMG signals acquired using bipolar electrode configuration is unfeasible at high contraction forces due to an excessive superimposition of MUAPs [3]. This electrophysiological limitation restricts our ability to use surface MUAP waveforms jointly with EMG interference pattern signals in our analysis. Nevertheless, Farina and colleagues [39] have previously shown that the volume conductor (e.g., layers skin and fat layers) intrinsic to surface EMG recordings restricts the morphological diversity of surface MUAP waveforms even for late recruited motor units, i.e., when the contraction intensity is increased. Therefore, our findings may extrapolate, to a certain degree, spectral characteristics of surface EMG signals acquired during isometric constant-force contractions at intensities somewhat above 20% of MVC.”

Furthermore, we have also added a consideration at the end of our Introduction section:

“Moreover, considering that the morphologies of surface MUAPs determine spectral characteristics of surface EMG signals (D. Farina, 2004; Dario Farina, Merletti, & Enoka, 2014; Rodriguez-Carreno, Gila-Useros, & Malanda-Trigueros, 2012), we expect consistency between the MDF and duration values estimated from MUAP waveforms with MDF values estimated from EMG interference pattern signals across different leg muscles. Considering electrophysiological constraints in estimating surface MUAP waveforms at high contraction intensities (Merletti & Farina, 2016), we have limited our analysis to low contraction forces (i.e., up to 20% of maximum force) for enabling that surface MUAP and EMG interference pattern signals to be jointly used to test this hypothesis.”

**References:**

Merletti, R., & Farina, D. (2016). Surface Electromyography: Physiology, Engineering, and Applications. (R. Merletti & D. Farina,Eds.), Surface Electromyography: Physiology, Engineering and Applications. Hoboken, New Jersey: John Wiley & Sons, Inc. Retrieved from https://doi.org/10.1002/9781119082934

Enoka, R. M., & Duchateau, J. (2017). Rate Coding and the Control of Muscle Force. Cold Spring Harbor Perspectives in Medicine, 7(10). Retrieved from https://doi.org/10.1101/cshperspect.a029702

Farina, D., Negro, F., Gazzoni, M., & Enoka, R. M. (2008). Detecting the Unique Representation of Motor-Unit Action Potentials in the Surface Electromyogram. Journal of Neurophysiology. Retrieved from https://doi.org/10.1152/jn.90219.2008

Comment 2:	*While the study proposes potential applications for the dataset in computational model validation, it would benefit from a discussion on the specific aspects or parameters that the dataset could effectively validate in these models.*

Thank you for your comment. The dataset available from this study includes a set of MUAP waveforms and EMG interference patterns signals which can be used for development and/or validation of computational models. Specifically for the **development** of phenomenological computational models of surface EMG signals, the time-series data of surface MUAP waveforms can be directly summed at time instants corresponding to motor unit discharges. The available time-series surface MUAP waveforms may also be expanded in Hermite-Rodriguez functions (Lo Conte, Merletti, & Sandri, 1994) for implementation in EMG simulations, consistent with the approach used by Farina and colleagues for simulation of intramuscular MUAP waveforms in (D. Farina, Crosetti, & Merletti, 2001). Furthermore, other types of waveforms, such as finite support wavelets, can be adjusted using the durations and other morphologic properties, such as number of peaks and amplitudes, estimated from the MUAP waveforms of our dataset (de Freitas & Kohn, 2020).

For **validation** of computational models, the available dataset of surface EMG interference pattern can be used to confirm stochastic properties of the simulated signals. For instance, the spectral components estimated from EMG signals of our dataset and simulated EMG signals may be compared using statistical methods (Bendat & Piersol, 2010). As an alternative, the median spectral frequency estimated the from EMG signals of our dataset, summarized in Fig 5, may also be compared. Additionally, MUAP waveforms simulated through volume conductor models (Ghosh, Kumar, Arjunan, Siddiqi, & Swaminathan, 2017; Teklemariam, Hodson-Tole, Reeves, Costen, & Cooper, 2016) can also be validated with morphologic properties estimated from our MUAP waveform dataset.

It is noteworthy that the available dataset of surface MUAP waveforms and EMG interference pattern signals is separated among muscles (Soleus, Medial Gastrocnemius, Lateral Gastrocnemius and Tibialis Anterior), each showing significantly different spectral components, as shown in this current work. Therefore, our dataset also enables means for the development and validation of computational models for specific leg muscles, which is critical for inferring appropriate interpretations from simulated surface myoelectric signals (Baker & Lemon, 1998; Mezzarane, Elias, Magalhes, Chaud, & Kohn, 2013; Moezzi et al., 2018; Wang, Stefano, & Allen, 2006).

We agree with the reviewer that specific aspects and parameters of our dataset for effectively validating computational models must be included in the manuscript for a better understanding of the advantages of its use. Therefore, we have added the following considerations to the Discussion section (4.3 Simulation and validation of surface EMG interference pattern signals):

“We showed that surface EMG signals acquired from TA muscle have more spectral components in lower frequencies compared with the plantar flexor muscles (SO, MG, LG, and TA), whereas their surface MUAP waveforms had longer durations. These results indicate that characteristics of myoelectric signals acquired from specific muscles should be taken into account for realistic simulations of surface EMGs, which may provide adequate parallels between computational simulation results and experimental data [12–15]. The experimental values estimated in this study (Figs 4 and 5) could help the development and validation of computational models of surface EMG of ankle plantar flexor and dorsiflexor muscles. For the validation of computational models, the MDF values shown in Fig 5 could be used as a reference for confirming spectral components of surface EMG signals simulated, for example, with volume conduction models [27]. Alternatively, the dataset of surface EMG interference pattern signals obtained in this study (available for download in https://doi.org/10.17605/OSF.IO/MXTQG) could be used to statistically validate the spectra estimated from EMG signals simulated with a model under development by proper time series analysis methods [43]. Importantly, the same configuration of electrodes used herein for recording surface EMG signals (see Section 2.2) should be employed in future experimental studies for enabling validations. For the development of phenomenological computational models of surface EMG signals, the dataset of surface MUAP waveforms obtained in this study (available for download in https://doi.org/10.17605/OSF.IO/MXTQG) could be directly summed at time instants corresponding to motor unit discharges. Alternatively, the time-series data of surface MUAP waveforms can be expanded in Hermite-Rodriguez functions, consistent with the approach used in [44] for intramuscular EMG signals. Finite support wavelets can also be simulated as MUAP waveforms by adjusting their morphologic properties, such as duration, number of peaks, and amplitudes, estimated from the MUAP waveforms of our dataset [24]. Moreover, surface EMG signals can also be simulated as the temporal sum of MUAP waveforms available herein for each muscle, instead of mathematically modeled waveforms, thus avoiding approximations stemming from the mathematical modeling [10,15].”

**References:**

Lo Conte, L. R., Merletti, R., & Sandri, G. V. (1994). Hermite Expansions of Compact Support Waveforms: Applications to Myoelectric Signals. IEEE Transactions on Biomedical Engineering, 41(12), 1147–1159. Retrieved from https://doi.org/10.1109/10.335863

Farina, D., Crosetti, A., & Merletti, R. (2001). A model for the generation of synthetic intramuscular EMG signals to test decomposition algorithms. IEEE Transactions on Biomedical Engineering. Retrieved from https://doi.org/10.1109/10.900250

de Freitas, R. M., & Kohn, A. F. (2020). Quantitative and Qualitative Parameters of Myoelectric Signals for Computational Simulations of Human Surface Electromyograms. In ACM International Conference Proceeding Series. Retrieved from https://doi.org/10.1145/3397391.3397446

Bendat, J. S., & Piersol, A. G. (2010). Random Data. Wiley Series in Probabillity and Statistics. Retrieved from https://doi.org/10.1002/9781118032428

Ghosh, D. M., Kumar, D., Arjunan, S. P., Siddiqi, A., & Swaminathan, R. (2017). A computational model to investigate the effect of pennation angle on surface electromyogram of Tibialis Anterior. PLoS ONE. Retrieved from https://doi.org/10.1371/journal.pone.0189036

Teklemariam, A., Hodson-Tole, E. F., Reeves, N. D., Costen, N. P., & Cooper, G. (2016). A finite element model approach to determine the influence of electrode design and muscle architecture on myoelectric signal properties. PLoS ONE. Retrieved from https://doi.org/10.1371/journal.pone.0148275

Baker, S. N., & Lemon, R. N. (1998). Computer simulation of post-spike facilitation in spike-triggered averages of rectified EMG. Journal of Neurophysiology. Retrieved from https://doi.org/10.1152/jn.1998.80.3.1391

Mezzarane, R. A., Elias, L. A., Magalhes, F. H., Chaud, V. M., & Kohn, A. F. (2013). Experimental and Simulated EMG Responses in the Study of the Human Spinal Cord. In Electrodiagnosis in New Frontiers of Clinical Research. InTech. Retrieved from https://doi.org/10.5772/54870

Moezzi, B., Schaworonkow, N., Plogmacher, L., Goldsworthy, M. R., Hordacre, B., McDonnell, M. D., … Triesch, J. (2018). Simulation of electromyographic recordings following transcranial magnetic stimulation. Journal of Neurophysiology. Retrieved from https://doi.org/10.1152/jn.00626.2017

Wang, W., Stefano, A. De, & Allen, R. (2006). A simulation model of the surface EMG signal for analysis of muscle activity during the gait cycle. Computers in Biology and Medicine. Retrieved from https://doi.org/10.1016/j.compbiomed.2005.04.002

Comment 3:	*The study hints at potential influences on MUAP waveform characteristics, such as muscle fiber conduction velocities. A more in-depth discussion on the impact of muscle fiber characteristics and electrode positioning would strengthen the interpretation of results.*

Thank you for your comment. Although there could exist multiple confounding factors that could explain the differences found across the EMG spectra from different muscles (Fig 4 and 5), some considerations about the impact of the remarkable muscle fiber characteristics and the electrode positioning can be highlighted. Leg muscles are distinctly structured by muscle fibers with different electrical and anatomical properties arranged with different pennation angles (Enoka, 2003). In turn, the relative position between the surface EMG electrodes and the anatomical position of muscle fibers determines spectral and morphological properties of surface myoelectric signals (Merletti & Farina, 2016; Rodriguez-Carreno et al., 2012). In our study, the Soleus muscle, located beneath the Gastrocnemii and attached to the Achilles tendon, is at a further distance from the recording electrodes compared to the lateral head of the Gastrocnemius muscle (Mezzarane & Kohn, 2002). Moreover, muscle fibers of the Soleus have shorter lengths and larger pennation angles compared with the Lateral Gastrocnemius muscle (Chow et al., 2000; Maganaris, Baltzopoulos, & Sargeant, 1998). These anatomical distinctions could account for the differences found between the spectral quantifiers of EMG interference pattern signals recorded from Soleus and Lateral Gastrocnemius muscles as force contraction increases (Fig 5). Furthermore, electrical properties of muscle determine the conduction velocity of action potentials along muscle fibers, while affecting the duration of surface MUAP waveforms (Rodriguez-Carreno et al., 2012). It has been previously shown that the conduction velocities of muscle fibers of Soleus muscle are higher compared with Tibialis Anterior muscle (Jensen, Manresa, Frahm, & Andersen, 2013). These results could explain the difference in the duration and spectral components that were found between surface MUAP waveforms estimated from Soleus and Tibialis Anterior muscles.

We agree with the reviewer that these points should be discussed in out manuscript to allow for better interpretation of our results. Therefore, we have added the following considerations to the Discussion section (4.1 Spectral content of surface MUAP waveforms):

“It is noteworthy that the duration of surface MUAP waveforms may be affected by electrical properties of muscle fibers, such as the conduction velocity of action potentials along muscle fibers [26]. These results indicate that muscle fibers of the TA muscle might have lower conduction velocities compared with the other plantar flexor muscles (SO, MG, and LG), as suggested by [42]. Nevertheless, there could exist multiple confounding factors, such as the relative position of muscle fibers and the recording electrodes, that could explain the morphological characteristics of surface MUAP waveforms [16].”

Furthermore, we have also modified the section “4.2 Spectral content of surface EMG interference pattern signals” as follows:

“It is thus possible that a change in fiber pennation angle and length with increasing isometric contraction force has resulted in the spectral content of myoelectric signals obtained from LG shifting towards higher frequencies [30]. In fact, MDF values seem to increase with increasing contraction intensities for all muscles in Fig 5, despite not being a statistically significant difference. It is also noteworthy that the Soleus muscle, located beneath the Gastrocnemii and attached to the Achilles tendon, is at a further distance from the recording electrodes compared to the lateral head of the Gastrocnemius muscle [32]. The relative distance between the surface EMG electrodes and the anatomical position of the muscle fibers could also be a confounding factor in the results shown in Fig 5.”

**References:**

Enoka, R. M. (2003). Neuromechanics of movement. In Neuromechanics of movement. Retrieved from https://doi.org/10.1186/1744-9081-2-37

Merletti, R., & Farina, D. (2016). Surface Electromyography: Physiology, Engineering, and Applications. (R. Merletti & D. Farina,Eds.), Surface Electromyography: Physiology, Engineering and Applications. Hoboken, New Jersey: John Wiley & Sons, Inc. Retrieved from https://doi.org/10.1002/9781119082934

Rodriguez-Carreno, I., Gila-Useros, L., & Malanda-Trigueros, A. (2012). Motor Unit Action Potential Duration: Measurement and Significance. In Advances in Clinical Neurophysiology. InTech. Retrieved from https://doi.org/10.5772/50265

Mezzarane, R. A., & Kohn, A. F. (2002). Bilateral soleus H-reflexes in humans elicited by simultaneous trains of stimuli: symmetry, variability, and covariance. Journal of Neurophysiology, 87, 2074–2083. Retrieved from https://doi.org/10.1152/jn.00129.2001

Chow, R. S., Medri, M. K., Martin, D. C., Leekam, R. N., Agur, A. M., & McKee, N. H. (2000). Sonographic studies of human soleus and gastrocnemius muscle architecture: Gender variability. European Journal of Applied Physiology. Retrieved from https://doi.org/10.1007/s004210050677

Maganaris, C. N., Baltzopoulos, V., & Sargeant, A. J. (1998). In vivo measurements of the triceps surae complex architecture in man: implications for muscle function. The Journal of Physiology, 512(Pt 2), 603–14. Retrieved from https://doi.org/10.1111/j.1469-7793.1998.603be.x

Jensen, M. B., Manresa, J. A. B., Frahm, K. S., & Andersen, O. K. (2013). Analysis of muscle fiber conduction velocity enables reliable detection of surface EMG crosstalk during detection of nociceptive withdrawal reflexes. BMC Neuroscience. Retrieved from https://doi.org/10.1186/1471-2202-14-39

---------------------------------------------

**Responses to Editor:**

The authors would like to thank the Editor for the comments and considerations for meeting the journal requirements of submission.

Comment 1:	*Please ensure that your manuscript meets PLOS ONE's style requirements, including those for file naming.*

Thank you for your consideration. We have reviewed the PLOS ONE’s style requirements and adjusted the manuscript accordingly. We have modified the style of the title page and the manuscript body according to the PLOS ONE’s template sent by the Editor. We apologize for not meeting the style requirements in the initial submission.

Comment 2:	*Please note that PLOS ONE has specific guidelines on code sharing for submissions in which author-generated code underpins the findings in the manuscript. In these cases, all author-generated code must be made available without restrictions upon publication of the work.*

Thank you for your consideration. We have publicly shared our codes together with the dataset of surface motor unit action potentials and EMG interference pattern signals through OSF (<https://doi.org/10.17605/OSF.IO/MXTQG>). We made an effort to adhere to the best practices of code-sharing outlined by the PLOS ONE’s guidelines. We have also added to the Data Availability Statement that our code is publicly available through this link.

Comment 3:	*Please state what role the funders took in the study. If the funders had no role, please state: "The funders had no role in study design, data collection and analysis, decision to publish, or preparation of the manuscript." If this statement is not correct you must amend it as needed. Please include this amended Role of Funder statement in your cover letter; we will change the online submission form on your behalf.*

Thank you for your consideration. In fact, the funder had no role in the study design, data collection and analysis, decision to publish, or preparation of the manuscript. We have added this statement to our cover letter together with the funding-related information (Comment 4).

Comment 4:	*Please be informed that funding information should not appear in the Acknowledgments section or other areas of your manuscript. We will only publish funding information present in the Funding Statement section of the online submission form.
Please remove any funding-related text from the manuscript and let us know how you would like to update your Funding Statement. Currently, your Funding Statement reads as follows: "The Conselho Nacional de Desenvolvimento Científico e Tecnológico (CNPq) financed the scholarship of R.M.F. (Proc 130034/2017-7) during the development of this study. Moreover, A.F.K. has a fellowship from CNPq (# 311223/2021-4)"
Please include your amended statements within your cover letter; we will change the online submission form on your behalf.*

Thank you for your consideration. We have removed the funding-related statement mentioned by the Editor from our manuscript. We also have included this funding-related statement to our cover letter together with information about the role of the funders in our study (Comment 3).

Comment 5:	*We noted in your submission details that a portion of your manuscript may have been presented or published elsewhere:"Yes. The duration of motor unit action potentials have been used for a qualitative analysis in a conference: de Freitas RM, Kohn AF. Quantitative and Qualitative Parameters of Myoelectric Signals for Computational Simulations of Human Surface Electromyograms. In: ACM International Conference Proceeding Series. 2020."
Please clarify whether this [conference proceeding or publication] was peer-reviewed and formally published. If this work was previously peer-reviewed and published, in the cover letter please provide the reason that this work does not constitute dual publication and should be included in the current manuscript.*

Thank you for your consideration. As a subcomponent of the present study, we have analyzed a data series of decomposed motor unit action potential (MUAP) waveforms previously presented in a conference proceeding: *"de Freitas RM, Kohn AF. Quantitative and Qualitative Parameters of Myoelectric Signals for Computational Simulations of Human Surface Electromyograms. In: ACM International Conference Proceeding Series. 2020."*. The conference proceeding was peer-reviewed and published with the DOI <https://doi.org/10.1145/3397391.3397446>, which both authors own the copyright. We have included the following considerations to our cover letter:

***In this conference proceeding work***, we have characterized the dataset of MUAP waveforms using only time-domain parameters (e.g., amplitude, duration, and number of peaks). Statistical methods were not used for these characterizations. The aim of that work was to explore the differences across qualitative and quantitative characteristics of MUAP waveforms in different muscles. ***In the current study***, however, we characterized and compared this dataset of MUAP waveforms using time-domain (duration) and frequency-domain (power spectrum and spectral median frequency) approaches. Moreover, we have also analyzed the spectral components of a dataset of surface EMG interference pattern signals which have not been presented/published elsewhere. The inclusion of these EMG interference pattern signals imparts significantly more power to the present work than the restriction to isolated MUAP time-domain analyses as presented in the 2020 Conference paper. Importantly, our main object in this current work differs from the one previously presented while it aims to infer spectral differences of myoelectric signal (MUAPs and EMG interference patterns) across different muscles. Furthermore, we have processed and analyzed data series of MUAPs using different methods in this current work compared to previously. The statistical comparisons of the durations of MUAP waveforms across different muscles, as well as the estimation of spectral properties of MUAP waveforms presented in this work have not been published elsewhere. Overall, since different intellectual material, different approaches and different results are presented herein, we strongly believe that this would not constitute a dual publication.

Comment 6:	*Please remove your figures from within your manuscript file, leaving only the individual TIFF/EPS image files, uploaded separately. These will be automatically included in the reviewers’ PDF.*

Thank you for your consideration. We have removed our figures from within our manuscript file. Following the PLOS ONE’s style requirements, we kept only the captions of each figure after the paragraph where they are firstly mentioned. Moreover, individual EPS image files were uploaded separately.

Comment 7:	*Please review your reference list to ensure that it is complete and correct. If you have cited papers that have been retracted, please include the rationale for doing so in the manuscript text or remove these references and replace them with relevant current references. Any changes to the reference list should be mentioned in the rebuttal letter that accompanies your revised manuscript. If you need to cite a retracted article, indicate the article’s retracted status in the References list and also include a citation and full reference for the retraction notice.*

Thank you for your consideration. We have reviewed the reference list. We confirm that the reference list is complete and correct.
