# Supplementary material for: Spectral characterization of human leg EMG signals from an open access dataset for the development of computational models
Source: PLoS One. 2024 Apr 29;19(4):e0302632. doi: 10.1371/journal.pone.0302632 (PMC11057972; doi:10.1371/journal.pone.0302632)
Supplement: Peer review history

# Peer review: Decision Letter 0

**DOI:** 10.1371/journal.pone.0302632.r001

**Authors:** Emiliano Cè

23 Jan 2024

PONE-D-23-42461Spectral characterization of human leg EMG signals from an open access dataset for the development of computational modelsPLOS ONE

Dear Dr. de Freitas,

Thank you for submitting your manuscript to PLOS ONE. After careful consideration, we feel that it has merit but does not fully meet PLOS ONE’s publication criteria as it currently stands. Therefore, we invite you to submit a revised version of the manuscript that addresses the points raised during the review process.

 Please submit your revised manuscript by Mar 08 2024 11:59PM. If you will need more time than this to complete your revisions, please reply to this message or contact the journal office at plosone@plos.org. When you're ready to submit your revision, log on to https://www.editorialmanager.com/pone/ and select the 'Submissions Needing Revision' folder to locate your manuscript file.

Please include the following items when submitting your revised manuscript:

- A rebuttal letter that responds to each point raised by the academic editor and reviewer(s). You should upload this letter as a separate file labeled 'Response to Reviewers'.
- A marked-up copy of your manuscript that highlights changes made to the original version. You should upload this as a separate file labeled 'Revised Manuscript with Track Changes'.
- An unmarked version of your revised paper without tracked changes. You should upload this as a separate file labeled 'Manuscript'.

If you would like to make changes to your financial disclosure, please include your updated statement in your cover letter. Guidelines for resubmitting your figure files are available below the reviewer comments at the end of this letter.

If applicable, we recommend that you deposit your laboratory protocols in protocols.io to enhance the reproducibility of your results. Protocols.io assigns your protocol its own identifier (DOI) so that it can be cited independently in the future. For instructions see: https://journals.plos.org/plosone/s/submission-guidelines#loc-laboratory-protocols. Additionally, PLOS ONE offers an option for publishing peer-reviewed Lab Protocol articles, which describe protocols hosted on protocols.io. Read more information on sharing protocols at https://plos.org/protocols?utm_medium=editorial-email&utm_source=authorletters&utm_campaign=protocols.

We look forward to receiving your revised manuscript.

Kind regards,

Emiliano Cè

Academic Editor

PLOS ONE

Journal Requirements:

When submitting your revision, we need you to address these additional requirements.

1. Please ensure that your manuscript meets PLOS ONE's style requirements, including those for file naming. The PLOS ONE style templates can be found at 

https://journals.plos.org/plosone/s/file?id=wjVg/PLOSOne_formatting_sample_main_body.pdf and 

https://journals.plos.org/plosone/s/file?id=ba62/PLOSOne_formatting_sample_title_authors_affiliations.pdf

2. Please note that PLOS ONE has specific guidelines on code sharing for submissions in which author-generated code underpins the findings in the manuscript. In these cases, all author-generated code must be made available without restrictions upon publication of the work. Please review our guidelines at https://journals.plos.org/plosone/s/materials-and-software-sharing#loc-sharing-code and ensure that your code is shared in a way that follows best practice and facilitates reproducibility and reuse.

3. Thank you for stating the following financial disclosure: 

"The Conselho Nacional de Desenvolvimento Científico e Tecnológico (CNPq) financed the scholarship of R.M.F. (Proc 130034/2017-7) during the development of this study. Moreover, A.F.K. has a fellowship from CNPq (# 311223/2021-4)"

Please state what role the funders took in the study.  If the funders had no role, please state: "The funders had no role in study design, data collection and analysis, decision to publish, or preparation of the manuscript." If this statement is not correct you must amend it as needed. 

Please include this amended Role of Funder statement in your cover letter; we will change the online submission form on your behalf.

4. Thank you for stating the following in the Acknowledgments Section of your manuscript: 

"We are grateful to Felipe Fava de Lima for the technical assistance during data acquisition, and to all participants who were subjects in this study. We acknowledge the Conselho Nacional de Desenvolvimento Científico e Tecnológico (CNPq) for financing a scholarship to R.M.F. (Proc 130034/2017-7) during the development of this study. A.F.K. has a fellowship from CNPq (#311223/2021-4)"

Please be informed that funding information should not appear in the Acknowledgments section or other areas of your manuscript. We will only publish funding information present in the Funding Statement section of the online submission form. 

Please remove any funding-related text from the manuscript and let us know how you would like to update your Funding Statement. Currently, your Funding Statement reads as follows: 

"The Conselho Nacional de Desenvolvimento Científico e Tecnológico (CNPq) financed the scholarship of R.M.F. (Proc 130034/2017-7) during the development of this study. Moreover, A.F.K. has a fellowship from CNPq (# 311223/2021-4)"

Please include your amended statements within your cover letter; we will change the online submission form on your behalf.

5. We noted in your submission details that a portion of your manuscript may have been presented or published elsewhere:

"Yes. The duration of motor unit action potentials have been used for a qualitative analysis in a conference:

de Freitas RM, Kohn AF. Quantitative and Qualitative Parameters of Myoelectric Signals for Computational Simulations of Human Surface Electromyograms. In: ACM International Conference Proceeding Series. 2020."

Please clarify whether this [conference proceeding or publication] was peer-reviewed and formally published. If this work was previously peer-reviewed and published, in the cover letter please provide the reason that this work does not constitute dual publication and should be included in the current manuscript.

6. Please remove your figures from within your manuscript file, leaving only the individual TIFF/EPS image files, uploaded separately. These will be automatically included in the reviewers’ PDF.

7. Please review your reference list to ensure that it is complete and correct. If you have cited papers that have been retracted, please include the rationale for doing so in the manuscript text, or remove these references and replace them with relevant current references. Any changes to the reference list should be mentioned in the rebuttal letter that accompanies your revised manuscript. If you need to cite a retracted article, indicate the article’s retracted status in the References list and also include a citation and full reference for the retraction notice.

**Additional Editor Comments:**

Dear Authors,

Your manuscript has been reviewed by an expert in the filed who reported some minor issues you should consider while revisiting the work. 

[Note: HTML markup is below. Please do not edit.]

Reviewers' comments:

Reviewer's Responses to Questions

**Comments to the Author**

1. Is the manuscript technically sound, and do the data support the conclusions?

The manuscript must describe a technically sound piece of scientific research with data that supports the conclusions. Experiments must have been conducted rigorously, with appropriate controls, replication, and sample sizes. The conclusions must be drawn appropriately based on the data presented. 

Reviewer #1: Yes

**********

2. Has the statistical analysis been performed appropriately and rigorously? 

Reviewer #1: Yes

**********

3. Have the authors made all data underlying the findings in their manuscript fully available?

The PLOS Data policy requires authors to make all data underlying the findings described in their manuscript fully available without restriction, with rare exception (please refer to the Data Availability Statement in the manuscript PDF file). The data should be provided as part of the manuscript or its supporting information, or deposited to a public repository. For example, in addition to summary statistics, the data points behind means, medians and variance measures should be available. If there are restrictions on publicly sharing data—e.g. participant privacy or use of data from a third party—those must be specified.

Reviewer #1: Yes

**********

4. Is the manuscript presented in an intelligible fashion and written in standard English?

PLOS ONE does not copyedit accepted manuscripts, so the language in submitted articles must be clear, correct, and unambiguous. Any typographical or grammatical errors should be corrected at revision, so please note any specific errors here.

Reviewer #1: Yes

**********

5. Review Comments to the Author

Please use the space provided to explain your answers to the questions above. You may also include additional comments for the author, including concerns about dual publication, research ethics, or publication ethics. (Please upload your review as an attachment if it exceeds 20,000 characters)

Reviewer #1: The study provides a detailed investigation into the spectral characteristics of motor unit action potentials (MUAP) obtained from surface electromyography (EMG) signals in various leg muscles during weak contractions. Additionally, the research explores the spectral content of EMG interference patterns in response to isometric constant-force dorsiflexion and plantarflexion contractions at different intensities. The findings are crucial for understanding the myoelectric signals of leg muscles, with implications for the development and validation of computational models.The study involves a well-described experimental setup, including participant details, electrode configurations, and data acquisition procedures. The inclusion of fine wire EMG signals adds depth to the investigation. A thorough explanation of the data analysis methods is provided, including the estimation of surface MUAP waveforms, spectrum median frequency calculations, and fatigue effects analysis. The use of EMGLAB software and the availability of the dataset for future use enhance the reproducibility of the study. The statistical methods employed are suitable for the study design.

Minor Revisions:

1) The authors acknowledge the limitation in not analyzing surface MUAP waveforms and EMG interference pattern signals for contraction intensities above 20% of maximum voluntary contraction (MVC). I recommend the authors to better explain the reasons behind this choice.

2) While the study proposes potential applications for the dataset in computational model validation, it would benefit from a discussion on the specific aspects or parameters that the dataset could effectively validate in these models.

3) The study hints at potential influences on MUAP waveform characteristics, such as muscle fiber conduction velocities. A more in-depth discussion on the impact of muscle fiber characteristics and electrode positioning would strengthen the interpretation of results.

**********

6. PLOS authors have the option to publish the peer review history of their article (what does this mean?). If published, this will include your full peer review and any attached files.

If you choose “no”, your identity will remain anonymous but your review may still be made public.

**Do you want your identity to be public for this peer review?** For information about this choice, including consent withdrawal, please see our Privacy Policy.

Reviewer #1: No

**********

[NOTE: If reviewer comments were submitted as an attachment file, they will be attached to this email and accessible via the submission site. Please log into your account, locate the manuscript record, and check for the action link "View Attachments". If this link does not appear, there are no attachment files.]

While revising your submission, please upload your figure files to the Preflight Analysis and Conversion Engine (PACE) digital diagnostic tool, https://pacev2.apexcovantage.com/. PACE helps ensure that figures meet PLOS requirements. To use PACE, you must first register as a user. Registration is free. Then, login and navigate to the UPLOAD tab, where you will find detailed instructions on how to use the tool. If you encounter any issues or have any questions when using PACE, please email PLOS at figures@plos.org. Please note that Supporting Information files do not need this step.

# Author comment: Author response to Decision Letter 0

**DOI:** 10.1371/journal.pone.0302632.r002

9 Feb 2024

Responses to Reviewers: Revision 1 (R1) for ‘PONE-D-23-42461’ to the PLOS ONE – February 2024

The authors would like to thank the Editor and the Reviewer 1 for their constructive comments and suggestions. They helped us improve our manuscript considerably and meet the journal requirements for submission. Responses addressing specific comments from the Editor and Reviewer 1 are outlined below:

---------------------------------------------

Responses to Reviewer #1:

The authors would like to thank the Reviewer 1 for the constructive comments and suggestions. 

Comment 1: The authors acknowledge the limitation in not analyzing surface MUAP waveforms and EMG interference pattern signals for contraction intensities above 20% of maximum voluntary contraction (MVC). I recommend the authors to better explain the reasons behind this choice.

Thank you for your comment. Our main objective in this study was to compare the spectra of surface myoelectric signals acquired from different leg muscles using both motor unit action potential (MUAP) waveforms and EMG interference pattern signals. For enabling that MUAP waveforms and EMG interference pattern signals could be jointly used in our analysis to support our findings, we have limited our analysis to myoelectric signals acquired at low contraction intensities, i.e., 20% of the maximum voluntary contraction (MVC) force. Specifically, it is noteworthy that the estimation of surface MUAP waveforms from EMG signals acquired using bipolar electrode configuration is unfeasible at high contraction forces (Merletti & Farina, 2016). As the contraction force is increased, the number of recruited motor units and their discharge frequencies (i.e., rate coding) are increased (Enoka & Duchateau, 2017). At these high contraction forces, surface EMG signals are composed of an excessive sum of MUAPs which are not able to be discriminated. Therefore, considering these limiting constraints for estimating surface MUAP waveforms at high contraction forces and our objectives in this study, we have limited our analysis to surface myoelectric signals acquired at contraction forces below 20% MVC. Nevertheless, as we discuss in the Discussion section of our manuscript (4.4 Limitations), the volume conductor intrinsic to surface EMG recordings restricts the morphological diversity of surface MUAP waveforms even for late recruited motor units (D. Farina, Negro, Gazzoni, & Enoka, 2008). Thus, our findings may extrapolate to spectral characteristics of myoelectric EMG signals acquired at contraction forces somewhat above 20% MVC.

The authors acknowledge that these technical constraints must be added to the manuscript for better understanding of the limitations of the study. We have, therefore, added the following considerations to the Discussion section (4.4 Limitations):

 " A limitation of this study is that we did not analyze surface MUAP waveforms and EMG interference pattern signals for contraction intensities above 20% of MVC, which may limit the interpretation of our findings for signals acquired at stronger force levels. It is noteworthy that the estimation of surface MUAP waveforms from EMG signals acquired using bipolar electrode configuration is unfeasible at high contraction forces due to an excessive superimposition of MUAPs [3]. This electrophysiological limitation restricts our ability to use surface MUAP waveforms jointly with EMG interference pattern signals in our analysis. Nevertheless, Farina and colleagues [39] have previously shown that the volume conductor (e.g., layers skin and fat layers) intrinsic to surface EMG recordings restricts the morphological diversity of surface MUAP waveforms even for late recruited motor units, i.e., when the contraction intensity is increased. Therefore, our findings may extrapolate, to a certain degree, spectral characteristics of surface EMG signals acquired during isometric constant-force contractions at intensities somewhat above 20% of MVC.”

Furthermore, we have also added a consideration at the end of our Introduction section: 

“Moreover, considering that the morphologies of surface MUAPs determine spectral characteristics of surface EMG signals (D. Farina, 2004; Dario Farina, Merletti, & Enoka, 2014; Rodriguez-Carreno, Gila-Useros, & Malanda-Trigueros, 2012), we expect consistency between the MDF and duration values estimated from MUAP waveforms with MDF values estimated from EMG interference pattern signals across different leg muscles. Considering electrophysiological constraints in estimating surface MUAP waveforms at high contraction intensities (Merletti & Farina, 2016), we have limited our analysis to low contraction forces (i.e., up to 20% of maximum force) for enabling that surface MUAP and EMG interference pattern signals to be jointly used to test this hypothesis.”

References: 

Merletti, R., & Farina, D. (2016). Surface Electromyography: Physiology, Engineering, and Applications. (R. Merletti & D. Farina,Eds.), Surface Electromyography: Physiology, Engineering and Applications. Hoboken, New Jersey: John Wiley & Sons, Inc. Retrieved from https://doi.org/10.1002/9781119082934

Enoka, R. M., & Duchateau, J. (2017). Rate Coding and the Control of Muscle Force. Cold Spring Harbor Perspectives in Medicine, 7(10). Retrieved from https://doi.org/10.1101/cshperspect.a029702

Farina, D., Negro, F., Gazzoni, M., & Enoka, R. M. (2008). Detecting the Unique Representation of Motor-Unit Action Potentials in the Surface Electromyogram. Journal of Neurophysiology. Retrieved from https://doi.org/10.1152/jn.90219.2008

Comment 2: While the study proposes potential applications for the dataset in computational model validation, it would benefit from a discussion on the specific aspects or parameters that the dataset could effectively validate in these models.

Thank you for your comment. The dataset available from this study includes a set of MUAP waveforms and EMG interference patterns signals which can be used for development and/or validation of computational models. Specifically for the development of phenomenological computational models of surface EMG signals, the time-series data of surface MUAP waveforms can be directly summed at time instants corresponding to motor unit discharges. The available time-series surface MUAP waveforms may also be expanded in Hermite-Rodriguez functions (Lo Conte, Merletti, & Sandri, 1994) for implementation in EMG simulations, consistent with the approach used by Farina and colleagues for simulation of intramuscular MUAP waveforms in (D. Farina, Crosetti, & Merletti, 2001). Furthermore, other types of waveforms, such as finite support wavelets, can be adjusted using the durations and other morphologic properties, such as number of peaks and amplitudes, estimated from the MUAP waveforms of our dataset (de Freitas & Kohn, 2020). 

For validation of computational models, the available dataset of surface EMG interference pattern can be used to confirm stochastic properties of the simulated signals. For instance, the spectral components estimated from EMG signals of our dataset and simulated EMG signals may be compared using statistical methods (Bendat & Piersol, 2010). As an alternative, the median spectral frequency estimated the from EMG signals of our dataset, summarized in Fig 5, may also be compared. Additionally, MUAP waveforms simulated through volume conductor models (Ghosh, Kumar, Arjunan, Siddiqi, & Swaminathan, 2017; Teklemariam, Hodson-Tole, Reeves, Costen, & Cooper, 2016) can also be validated with morphologic properties estimated from our MUAP waveform dataset.

It is noteworthy that the available dataset of surface MUAP waveforms and EMG interference pattern signals is separated among muscles (Soleus, Medial Gastrocnemius, Lateral Gastrocnemius and Tibialis Anterior), each showing significantly different spectral components, as shown in this current work. Therefore, our dataset also enables means for the development and validation of computational models for specific leg muscles, which is critical for inferring appropriate interpretations from simulated surface myoelectric signals (Baker & Lemon, 1998; Mezzarane, Elias, Magalhes, Chaud, & Kohn, 2013; Moezzi et al., 2018; Wang, Stefano, & Allen, 2006).

We agree with the reviewer that specific aspects and parameters of our dataset for effectively validating computational models must be included in the manuscript for a better understanding of the advantages of its use. Therefore, we have added the following considerations to the Discussion section (4.3 Simulation and validation of surface EMG interference pattern signals):

“We showed that surface EMG signals acquired from TA muscle have more spectral components in lower frequencies compared with the plantar flexor muscles (SO, MG, LG, and TA), whereas their surface MUAP waveforms had longer durations. These results indicate that characteristics of myoelectric signals acquired from specific muscles should be taken into account for realistic simulations of surface EMGs, which may provide adequate parallels between computational simulation results and experimental data [12–15]. The experimental values estimated in this study (Figs 4 and 5) could help the development and validation of computational models of surface EMG of ankle plantar flexor and dorsiflexor muscles. For the validation of computational models, the MDF values shown in Fig 5 could be used as a reference for confirming spectral components of surface EMG signals simulated, for example, with volume conduction models [27]. Alternatively, the dataset of surface EMG interference pattern signals obtained in this study (available for download in https://doi.org/10.17605/OSF.IO/MXTQG) could be used to statistically validate the spectra estimated from EMG signals simulated with a model under development by proper time series analysis methods [43]. Importantly, the same configuration of electrodes used herein for recording surface EMG signals (see Section 2.2) should be employed in future experimental studies for enabling validations. For the development of phenomenological computational models of surface EMG signals, the dataset of surface MUAP waveforms obtained in this study (available for download in https://doi.org/10.17605/OSF.IO/MXTQG) could be directly summed at time instants corresponding to motor unit discharges. Alternatively, the time-series data of surface MUAP waveforms can be expanded in Hermite-Rodriguez functions, consistent with the approach used in [44] for intramuscular EMG signals. Finite support wavelets can also be simulated as MUAP waveforms by adjusting their morphologic properties, such as duration, number of peaks, and amplitudes, estimated from the MUAP waveforms of our dataset [24]. Moreover, surface EMG signals can also be simulated as the temporal sum of MUAP waveforms available herein for each muscle, instead of mathematically modeled waveforms, thus avoiding approximations stemming from the mathematical modeling [10,15].”

References: 

Lo Conte, L. R., Merletti, R., & Sandri, G. V. (1994). Hermite Expansions of Compact Support Waveforms: Applications to Myoelectric Signals. IEEE Transactions on Biomedical Engineering, 41(12), 1147–1159. Retrieved from https://doi.org/10.1109/10.335863

Farina, D., Crosetti, A., & Merletti, R. (2001). A model for the generation of synthetic intramuscular EMG signals to test decomposition algorithms. IEEE Transactions on Biomedical Engineering. Retrieved from https://doi.org/10.1109/10.900250

de Freitas, R. M., & Kohn, A. F. (2020). Quantitative and Qualitative Parameters of Myoelectric Signals for Computational Simulations of Human Surface Electromyograms. In ACM International Conference Proceeding Series. Retrieved from https://doi.org/10.1145/3397391.3397446

Bendat, J. S., & Piersol, A. G. (2010). Random Data. Wiley Series in Probabillity and Statistics. Retrieved from https://doi.org/10.1002/9781118032428

Ghosh, D. M., Kumar, D., Arjunan, S. P., Siddiqi, A., & Swaminathan, R. (2017). A computational model to investigate the effect of pennation angle on surface electromyogram of Tibialis Anterior. PLoS ONE. Retrieved from https://doi.org/10.1371/journal.pone.0189036

Teklemariam, A., Hodson-Tole, E. F., Reeves, N. D., Costen, N. P., & Cooper, G. (2016). A finite element model approach to determine the influence of electrode design and muscle architecture on myoelectric signal properties. PLoS ONE. Retrieved from https://doi.org/10.1371/journal.pone.0148275

Baker, S. N., & Lemon, R. N. (1998). Computer simulation of post-spike facilitation in spike-triggered averages of rectified EMG. Journal of Neurophysiology. Retrieved from https://doi.org/10.1152/jn.1998.80.3.1391

Mezzarane, R. A., Elias, L. A., Magalhes, F. H., Chaud, V. M., & Kohn, A. F. (2013). Experimental and Simulated EMG Responses in the Study of the Human Spinal Cord. In Electrodiagnosis in New Frontiers of Clinical Research. InTech. Retrieved from https://doi.org/10.5772/54870

Moezzi, B., Schaworonkow, N., Plogmacher, L., Goldsworthy, M. R., Hordacre, B., McDonnell, M. D., … Triesch, J. (2018). Simulation of electromyographic recordings following transcranial magnetic stimulation. Journal of Neurophysiology. Retrieved from https://doi.org/10.1152/jn.00626.2017

Wang, W., Stefano, A. De, & Allen, R. (2006). A simulation model of the surface EMG signal for analysis of muscle activity during the gait cycle. Computers in Biology and Medicine. Retrieved from https://doi.org/10.1016/j.compbiomed.2005.04.002

Comment 3: The study hints at potential influences on MUAP waveform characteristics, such as muscle fiber conduction velocities. A more in-depth discussion on the impact of muscle fiber characteristics and electrode positioning would strengthen the interpretation of results.

Thank you for your comment. Although there could exist multiple confounding factors that could explain the differences found across the EMG spectra from different muscles (Fig 4 and 5), some considerations about the impact of the remarkable muscle fiber characteristics and the electrode positioning can be highlighted. Leg muscles are distinctly structured by muscle fibers with different electrical and anatomical properties arranged with different pennation angles (Enoka, 2003). In turn, the relative position between the surface EMG electrodes and the anatomical position of muscle fibers determines spectral and morphological properties of surface myoelectric signals (Merletti & Farina, 2016; Rodriguez-Carreno et al., 2012). In our study, the Soleus muscle, located beneath the Gastrocnemii and attached to the Achilles tendon, is at a further distance from the recording electrodes compared to the lateral head of the Gastrocnemius muscle (Mezzarane & Kohn, 2002). Moreover, muscle fibers of the Soleus have shorter lengths and larger pennation angles compared with the Lateral Gastrocnemius muscle (Chow et al., 2000; Maganaris, Baltzopoulos, & Sargeant, 1998). These anatomical distinctions could account for the differences found between the spectral quantifiers of EMG interference pattern signals recorded from Soleus and Lateral Gastrocnemius muscles as force contraction increases (Fig 5). Furthermore, electrical properties of muscle determine the conduction velocity of action potentials along muscle fibers, while affecting the duration of surface MUAP waveforms (Rodriguez-Carreno et al., 2012). It has been previously shown that the conduction velocities of muscle fibers of Soleus muscle are higher compared with Tibialis Anterior muscle (Jensen, Manresa, Frahm, & Andersen, 2013). These results could explain the difference in the duration and spectral components that were found between surface MUAP waveforms estimated from Soleus and Tibialis Anterior muscles. 

We agree with the reviewer that these points should be discussed in out manuscript to allow for better interpretation of our results. Therefore, we have added the following considerations to the Discussion section (4.1 Spectral content of surface MUAP waveforms):

“It is noteworthy that the duration of surface MUAP waveforms may be affected by electrical properties of muscle fibers, such as the conduction velocity of action potentials along muscle fibers [26]. These results indicate that muscle fibers of the 

Attachment Submitted filename: Response to Reviewers.docx

# Peer review: Decision Letter 1

**DOI:** 10.1371/journal.pone.0302632.r003

**Authors:** Emiliano Cè

5 Apr 2024

Spectral characterization of human leg EMG signals from an open access dataset for the development of computational models

PONE-D-23-42461R1

Dear Dr. de Freitas,

We’re pleased to inform you that your manuscript has been judged scientifically suitable for publication and will be formally accepted for publication once it meets all outstanding technical requirements.

Within one week, you’ll receive an e-mail detailing the required amendments. When these have been addressed, you’ll receive a formal acceptance letter and your manuscript will be scheduled for publication.

An invoice will be generated when your article is formally accepted. Please note, if your institution has a publishing partnership with PLOS and your article meets the relevant criteria, all or part of your publication costs will be covered. Please make sure your user information is up-to-date by logging into Editorial Manager at Editorial Manager® and clicking the ‘Update My Information' link at the top of the page. If you have any questions relating to publication charges, please contact our Author Billing department directly at authorbilling@plos.org.

If your institution or institutions have a press office, please notify them about your upcoming paper to help maximize its impact. If they’ll be preparing press materials, please inform our press team as soon as possible -- no later than 48 hours after receiving the formal acceptance. Your manuscript will remain under strict press embargo until 2 pm Eastern Time on the date of publication. For more information, please contact onepress@plos.org.

Kind regards,

Emiliano Cè

Academic Editor

PLOS ONE

Additional Editor Comments (optional):

Reviewers' comments:

Reviewer's Responses to Questions

**Comments to the Author**

1. If the authors have adequately addressed your comments raised in a previous round of review and you feel that this manuscript is now acceptable for publication, you may indicate that here to bypass the “Comments to the Author” section, enter your conflict of interest statement in the “Confidential to Editor” section, and submit your "Accept" recommendation.

Reviewer #1: All comments have been addressed

**********

2. Is the manuscript technically sound, and do the data support the conclusions?

The manuscript must describe a technically sound piece of scientific research with data that supports the conclusions. Experiments must have been conducted rigorously, with appropriate controls, replication, and sample sizes. The conclusions must be drawn appropriately based on the data presented. 

Reviewer #1: Yes

**********

3. Has the statistical analysis been performed appropriately and rigorously? 

Reviewer #1: Yes

**********

4. Have the authors made all data underlying the findings in their manuscript fully available?

The PLOS Data policy requires authors to make all data underlying the findings described in their manuscript fully available without restriction, with rare exception (please refer to the Data Availability Statement in the manuscript PDF file). The data should be provided as part of the manuscript or its supporting information, or deposited to a public repository. For example, in addition to summary statistics, the data points behind means, medians and variance measures should be available. If there are restrictions on publicly sharing data—e.g. participant privacy or use of data from a third party—those must be specified.

Reviewer #1: Yes

**********

5. Is the manuscript presented in an intelligible fashion and written in standard English?

PLOS ONE does not copyedit accepted manuscripts, so the language in submitted articles must be clear, correct, and unambiguous. Any typographical or grammatical errors should be corrected at revision, so please note any specific errors here.

Reviewer #1: Yes

**********

6. Review Comments to the Author

Please use the space provided to explain your answers to the questions above. You may also include additional comments for the author, including concerns about dual publication, research ethics, or publication ethics. (Please upload your review as an attachment if it exceeds 20,000 characters)

Reviewer #1: The authors have done a very nice job in revising the ms. All my comments and suggestions have been addressed and revised version of the ms is much improved. In my oppinion, the ms represents a nice contribution to the current literature and shall be accepted in its present form. I congratulate the authors for their care in preparing the revision.

**********

7. PLOS authors have the option to publish the peer review history of their article (what does this mean?). If published, this will include your full peer review and any attached files.

If you choose “no”, your identity will remain anonymous but your review may still be made public.

**Do you want your identity to be public for this peer review?** For information about this choice, including consent withdrawal, please see our Privacy Policy.

Reviewer #1: No

**********
